# Volterra dendritic stimulus processors and biophysical spike generators with intrinsic noise sources

**DOI:** 10.3389/fncom.2014.00095

**Published:** 2014-09-01

**Authors:** Aurel A. Lazar, Yiyin Zhou

**Affiliations:** Department of Electrical Engineering, Columbia UniversityNew York, NY, USA

**Keywords:** Volterra dendritic stimulus processors, biophysical spike generators, noise, neural encoding, neural decoding, functional identification, Hodgkin-Huxley neuron, phase response curve

## Abstract

We consider a class of neural circuit models with internal noise sources arising in sensory systems. The basic neuron model in these circuits consists of a dendritic stimulus processor (DSP) cascaded with a biophysical spike generator (BSG). The dendritic stimulus processor is modeled as a set of nonlinear operators that are assumed to have a Volterra series representation. Biophysical point neuron models, such as the Hodgkin-Huxley neuron, are used to model the spike generator. We address the question of how intrinsic noise sources affect the precision in encoding and decoding of sensory stimuli and the functional identification of its sensory circuits. We investigate two intrinsic noise sources arising (i) in the active dendritic trees underlying the DSPs, and (ii) in the ion channels of the BSGs. Noise in dendritic stimulus processing arises from a combined effect of variability in synaptic transmission and dendritic interactions. Channel noise arises in the BSGs due to the fluctuation of the number of the active ion channels. Using a stochastic differential equations formalism we show that encoding with a neuron model consisting of a nonlinear DSP cascaded with a BSG with intrinsic noise sources can be treated as generalized sampling with noisy measurements. For single-input multi-output neural circuit models with feedforward, feedback and cross-feedback DSPs cascaded with BSGs we theoretically analyze the effect of noise sources on stimulus decoding. Building on a key duality property, the effect of noise parameters on the precision of the functional identification of the complete neural circuit with DSP/BSG neuron models is given. We demonstrate through extensive simulations the effects of noise on encoding stimuli with circuits that include neuron models that are akin to those commonly seen in sensory systems, e.g., complex cells in V1.

## 1. Introduction

Intrinsic noise sources are diverse and appear on many levels of a neuronal system ranging from electrical to chemical noise sources (Faisal et al., 2008; Destexhe and Rudolph-Lilith, 2012) and from single cells to networks of neurons. At the cellular and subcellular level, variability in biochemical reactions leads to stochastic transduction processes (Song et al., 2012), and ion channel fluctuations (Neher and Sakmann, 1976; White et al., 1998) result in variability in spike generation and propagation (Faisal and Laughlin, 2007). At the network level, probabilistic quantal release of neurotransmitters (Katz, 1962), background synaptic activity (Destexhe et al., 2003; Jocobson et al., 2005) and variability in timing of spikes from presynaptic neurons (Faisal and Neishabouri, 2014) are sources of stochastic fluctuation of synaptic conductances (Destexhe et al., 2001) that are believed to have a major impact on spike time variability (Yarom and Hounsgaard, 2011).

The existence of sources of noise also leads to variability in the spike times even when neurons are subject to the same, repeated inputs (Calvin and Stevens, 1968; Berry et al., 1997; de Ruyter van Steveninck et al., 1997). Spikes are the primary form of carriers of information in the nervous system and their timing is thought to be relevant to the message neurons need to convey (Rieke et al., 1999). Therefore, the variability of spike timing may reduce or damage the information being transmitted. It is quite remarkable, however, that sensory systems manage to be very robust even if they are subject to interference due to noise. Visual and auditory systems are two examples in which the stimuli are highly time varying. These systems have been reported to convey information with high spike timing precision (Butts et al., 2007; Kayser et al., 2010).

Noise may be useful in facilitating signal detection (McDonnell and Ward, 2011). Still, interference due to noise poses an important limit on how well sensory systems can represent input stimuli. It is not clear how intrinsic noise sources affect the representation of sensory inputs based on spike times, and how they impact the functional identification of sensory neurons.

We study the representation of sensory stimuli using a novel neural circuit model, that extends previously proposed models (Lazar et al., 2010; Lazar and Slutskiy, 2014, in press) in terms of architectural complexity and the existence of intrinsic noise sources. Our base level circuit architecture consists of two interconnected neurons, each with two cascaded stages. The first stage comprises two types of dendritic stimulus processors. The first dendritic stimulus processor performs *nonlinear* processing of input stimuli in the feedforward path leading to the spike generator. The second dendritic stimulus processor performs *nonlinear* processing in the feedback loop whose inputs are spike trains generated by biophysical spike generators (BSGs). The BSGs constitute the second stage of the base level circuit.

Our nonlinear dendritic stimulus processors describe functional I/O relationships between the dendritic outputs in the first stage and inputs that are either sensory stimuli or spikes generated by BSGs. DSPs are modeled using Volterra series. Volterra series have been used for analyzing nonlinear neuronal responses in many contexts (Lu et al., 2011; Eikenberry and Marmarelis, 2012), and have been applied to the identification of single neurons in many of sensory areas (Benardete and Kaplan, 1997; Theunissen et al., 2000; Clark et al., 2011). Volterra dendritic processors can model a wide range of nonlinear effects commonly seen in sensory systems (Lazar and Slutskiy, in press). Here, in addition, we introduce nonlinear interactions between neurons in the feedback and cross-feedback paths. This gives rise to interesting neural processing capabilities directly in the spike domain, e.g., coincidence detection (Agmon-Snir et al., 1998; Stuart and Häusser, 2001). The relationships described here by the Volterra model are functional and do not address the underlying circuit/dendritic tree level interactions. However, the latter have recently been subject to intense investigations (London and Häusser, 2005; Wohrer and Kornprobst, 2009; Werblin, 2011; Xu et al., 2012; Yonehara et al., 2013; Zhang et al., 2013). Conductance-based, biophysical spike generators are well established models that have been extensively used in studies of neuronal excitability and in large simulations of spiking neural networks (Izhikevich, 2007). Following Lazar (2010), we use formal BSG models to represent sensory stimuli under noisy conditions.

We formulate the encoding, decoding and functional identification problems under the neural encoding framework of Time Encoding Machines (TEMs). In this modeling framework the exact timing of spikes is considered to carry information about input stimuli (Lazar and Tóth, 2004). The separation into dendritic stimulus processors and spike mechanisms mentioned above allows us to study synaptic inputs and spike generation mechanisms separately, and hence independently model the intrinsic noise sources of each component. We incorporate two important noise sources into a general single-input multi-output neural circuit model. The first is a channel noise source that arises in spike generation (White et al., 2000). The second is a synaptic noise source due to a variety of fluctuating synaptic currents (Manwani and Koch, 1999).

Based on the rigorous formalism of TEMs, we show how noise arising in dendritic stimulus processors and in biophysical spike generators is related to the measurement error in generalized sampling. Dendritic stimulus processing and spike generation can then be viewed as a generalized sampling scheme that neurons utilize to represent sensory inputs (Lazar et al., 2010). Contrary to traditional sampling where the signal amplitude is sampled at clock times, neurons asynchronously sample all stimuli.

We systematically investigate how the strength of noise sources degrades the faithfulness of stimulus representation and the quality of functional identification of our proposed class of neural circuits. Furthermore, since the representation is based on spike timing, it is natural to investigate how spike timing variability affects the precision in representing the amplitude information of sensory stimuli.

The work presented here requires a substantial amount of investment in the mathematical formalism employed throughout. There are a number of benefits in doing so, however. Formulating the problem of stimulus encoding with a neural circuit with intrinsic noise sources as one of generalized sampling, i.e., of taking noisy measurements is of interest to both experimentalists and theoreticians alike. Understanding that the problem of neural decoding and functional identification are dual to each other is key to building on either or both. Finding how many repeat experiments need to be performed for a precise quantitative identification of Volterra kernels is of great value in neurophysiology. A further qualitative insight of our work is that for neural circuits with arbitrary connectivity, feedforward kernels are typically easier to estimate than feedback kernels. Finally, our finding that some key nonlinear neural circuits are tractable for detailed noise analysis suggests a wide reaching analytical methodology.

## 2. Modeling nonlinear neural circuits, stimuli, and noise

We present in Section 2.1 the general architecture of the neural circuits considered in this paper. In Section 2.2 we discuss the modeling of the space of stimuli. Volterra DSPs are the object of Section 2.3. Finally, in Section 2.4 we provide models of BSGs with intrinsic noise sources.

### 2.1. Neural circuit architecture

**Figure 1:**
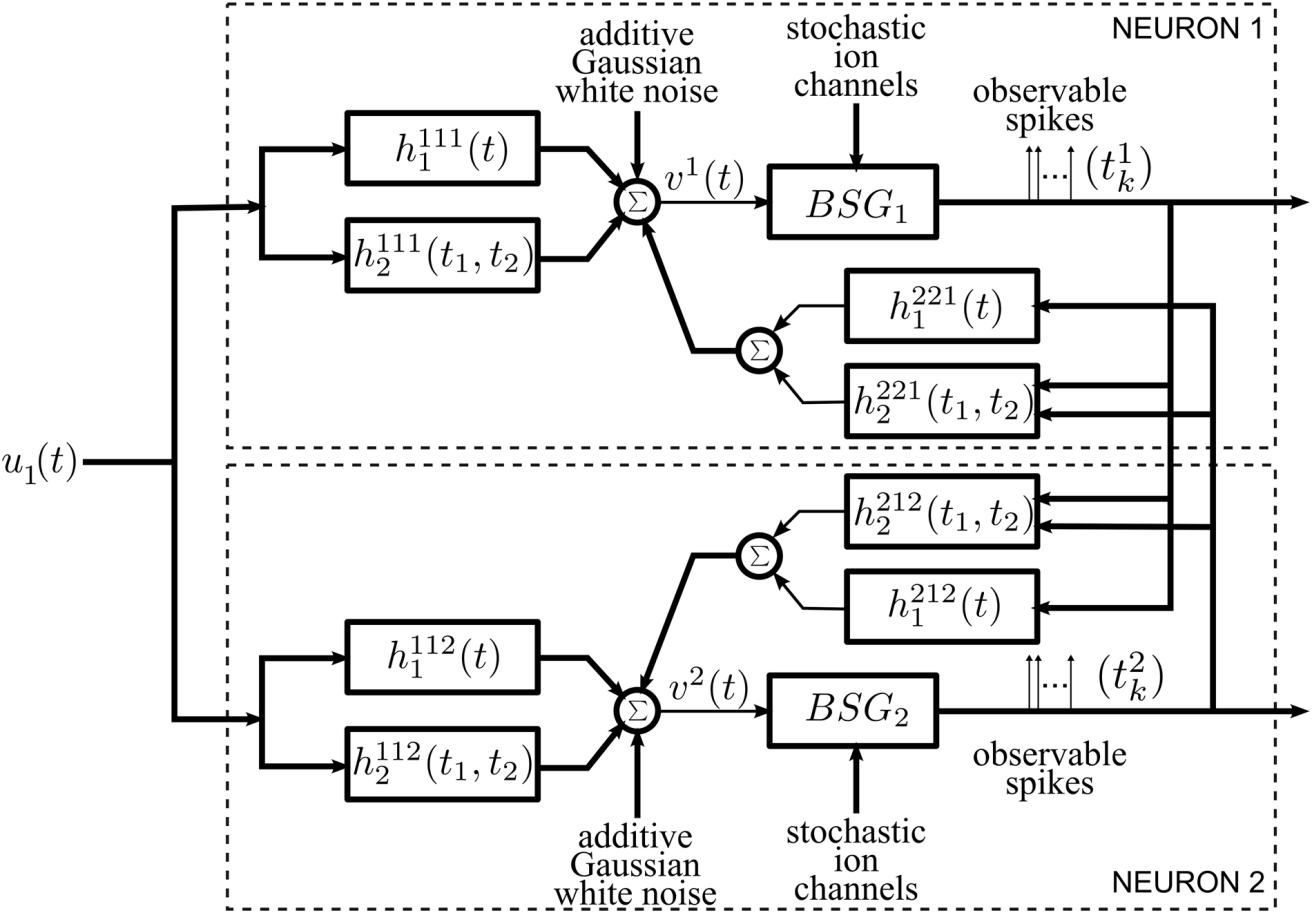
**Diagram of the architecture of the neural circuits**.

The general architecture of the neural circuit considered here is shown in simplified form in Figure 1. It consists of two neurons with a common time-varying input stimulus. With added notational complexity the neural circuit in Figure 1 can easily be extended in two ways. First, multiples of such circuits can encode a stimulus in parallel (see Section 2.1 in the Supplementary Material). In this case only pairs of neurons are interconnected through the feedback kernels. Second, more neurons can be considered in the neural circuit of Figure 1; all these neurons can be fully interconnected through feedback loops.

Each neuron *i*, *i* = 1, 2, receives a single time-varying input stimulus *u*_1_(*t*). The modeling of the input stimulus is discussed in Section 2.2. The output of each of the biophysical spike generators (BSGs) is a spike sequence denoted by (*t*^1^_*k*_) and (*t*^2^_*l*_), *k*, *l* ∈ ℤ.

The input stimulus *u*_1_(*t*) is first processed by a feedforward Dendritic Stimulus Processor (feedforward DSP) (Lazar and Slutskiy, in press). The feedforward DSP models the aggregated effect of processing in the neural circuits in the prior stages and in the dendritic tree of neuron *i* = 1, 2. For example, if the neurons in the model circuit are considered to be Retinal Ganglion Cells (RGCs), then the feedforward Volterra DSP models the processing that takes place in the outer- and inner-plexiform layers of the retina as well as in the dendritic trees of an RGC (Werblin, 2011; Masland, 2012). The feedforward DSPs are modeled here as second order Volterra expansion terms (Volterra, 1930). The first order terms *h*^11*i*^_1_(*t*) in the feedforward DSPs are linear filters typically used in modeling receptive fields. The second order terms *h*^11*i*^_2_(*t*_1_, *t*_2_) model nonlinear operations on the stimulus *u*_1_(*t*).

A second group of Volterra DSPs models the cross-feedback interactions between the two neurons. Instead of time-varying stimuli, the output spikes generated by the BSGs are the inputs to these DSPs. We therefore refer to these as feedback Dendritic Stimulus Processors (feedback DSPs). The output spikes of each individual neuron *i* are processed by the first order term *h*^2*ji*^_1_(*t*), *i*, *j* = 1, 2, *i* ≠ *j*. In addition, output spikes from both neurons interact nonlinearly through the second order terms *h*^2*ji*^_2_(*t*_1_, *t*_2_), *i*, *j* = 1, 2, *i* ≠ *j*. The summed responses from the first order feedback DSP *h*^2*ji*^_1_ and the second order feedback DSP *h*^2*ji*^_2_ are fed back to neuron *i* as additional dendritic currents.

The dendritic currents consisting of the output of the DSPs with added noise are subsequently encoded by biophysical spike generators. BSGs are biophysically realistic axon hillock spike generator models that are governed by a set of differential equations with multiple types of ion channels (Hodgkin and Huxley, 1952; Izhikevich, 2007). The detailed BSG models are introduced in Section 2.4. The spike times of output spikes generated by the BSGs are assumed to be observable.

We identify two intrinsic noise sources of the proposed neural circuit. First, the feedforward DSPs and the feedback DSPs are affected by additive Gaussian white noise. This noise arises from the combined effect along the path from sensory transduction to synaptic integration and includes synaptic background noise and stochasticity in the dendritic tree (Manwani and Koch, 1999; Fellous et al., 2003; Destexhe and Rudolph-Lilith, 2012). Since the outputs of the feedforward and feedback DSPs are additively combined, we consider, for simplicity, a single source of additive Gaussian white noise. Second, the ion channels of the BSGs are intrinsically stochastic and introduce noise in the spike generators (White et al., 2000; Hille, 2001).

### 2.2. Modeling signal spaces

Two signal spaces will be considered here. The first, models the space of input signals to feedforward DSPs. The second models the space of input spikes to feedback DSPs. These spaces will be formally described below.

#### 2.2.1. Modeling the space of input stimuli

We model the space of input stimuli as a Reproducing Kernel Hilbert Space (RKHS) (Berlinet and Thomas-Agnan, 2004). RKHSs are versatile vector spaces for modeling signals arising in computational neuroscience, signal processing and machine learning. For example, auditory signals, olfactory signals and visual signals can readily be modeled as band-limited functions of an RKHS with a sinc or Dirichlet kernel (Lazar et al., 2010; Lazar and Slutskiy, 2013). A particular choice of RKHSs in this article is the space of trigonometric polynomials. The computational advantage of working on the space of trignometric polynomials has been discussed (Lazar et al., 2010) and is closely related to the algorithmic tractability of the Fourier series in the digital domain. If the biological signals have unknown bandwidth with a spectrum that falls off fast enough, many Sobolev spaces might be a suitable choice of RKHS (Berlinet and Thomas-Agnan, 2004; Lazar and Pnevmatikakis, 2009). In such spaces the norm may include the derivative of the signal, i.e., the rate of change of the signal that many neurons are sensitive to Kim et al. (2011).

The space of trigonometric polynomials is defined as below.

**Definition 2.1**. *The space of trigonometric polynomials* 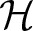
^1^_1_ *is a function space whose elements are functions defined on the domain* 𝔻_1_ = [0, *S*^1^], *S*^1^ ∈ ℝ_+_, *of the form*

(1) $$u_{1}(t)=\sum_{l=-L^{1}}^{L^{1}}u_{l}e_{l}(t),$$

where

(2) $$e_{l}(t)=\frac{1}{\sqrt{S^{1}}}e^{jl\frac{\Omega^{1}}{L^{1}}t},l=-L^{1},\cdots ,L^{1},$$

*are a set of orthonormal basis functions*. Ω^1^ *denotes the bandwidth and L*^1^ *is the order of the space*.


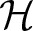
^1^_1_ endowed with the inner product:

(3) $$\langle u_{1},v_{1}\rangle =\int_{{𝔻}_{1}}u_{1}(t)\bar{v_{1}(t)}dt$$

is a Hilbert Space. Intuitively, the basis functions *e*_*l*_(*t*), *l* = −*L*^1^, …, *L*^1^, can be interpreted as a set of discrete spectral lines uniformly spaced in the frequency domain between −Ω^1^ and Ω^1^. For a given signal *u*_1_(*t*), the amplitude of its spectral lines is determined by the coefficients *u*_l_, *l* = −*L*^1^, …, *L*^1^.

**Remark 2.2**. *Functions in* 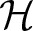
^1^_1_ *are periodic over* ℝ *with period* $S^{1}=\frac{2\pi L^{1}}{\Omega^{1}}$. *Therefore, the domain* 𝔻_1_ *covers exactly one period of the function. Note that the u*_*l*_'*s are closely related to the Fourier coefficients of the periodic signal u*_1_(*t*), *and can thereby be very efficiently computed via the Fast Fourier Transform*.


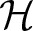
^1^_1_ is an RKHS with reproducing kernel (RK)

(4) $$K_{1}^{1}(t;s)=\sum_{l=-L^{1}}^{L^{1}}e_{l}(t-s).$$

It can be easily verified that the RK satisfies the reproducing property

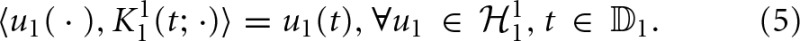



**Definition 2.3**. *We shall also consider the tensor product space* 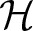
^1^_2_ *on the domain* 𝔻_2_ = [0, *S*^1^] × [0, *S*^1^], *whose elements are of the form*

(6) $$u_{2}(t_{1},t_{2})=\sum_{l_{1}=-L^{1}}^{L^{1}}\sum_{l_{2}=-L^{1}}^{L^{1}}u_{l_{1}l_{2}}e_{l_{1}l_{2}}(t_{1},t_{2}),$$

where

(7) $$e_{l_{1}l_{2}}(t_{1},t_{2})=\frac{1}{S^{1}}e^{jl_{1}\frac{\Omega^{1}}{L^{1}}t_{1}}e^{jl_{2}\frac{\Omega^{1}}{L^{1}}t_{2}},$$

*are a set of functions forming an orthonormal basis*.


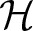
^1^_2_ is again an RKHS with RK

(8) $$K_{2}^{1}(t_{1},t_{2};s_{1},s_{2})=\sum_{l_{1}=-L^{1}}^{L^{1}}\sum_{l_{2}=-L^{1}}^{L^{1}}e_{l_{1}l_{2}}(t_{1}-s_{1},t_{2}-s_{2}).$$

Note that we use the subscript to indicate the dimension of the domain of functions, i.e., the number of variables the functions in the RKHS have, and use the superscript 1 to indicate the input space.

Projections of functions onto the RKHSs introduced here can be defined as follows:

**Definition 2.4**. *Let h*_1_ ∈ 𝕃^1^(𝔻_1_), *where* 𝕃^1^ *denotes the space of Lebesgue integrable functions. The operator* 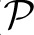
^1^: 𝕃^1^(𝔻_1_) → 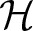
^1^_1_ *given by*

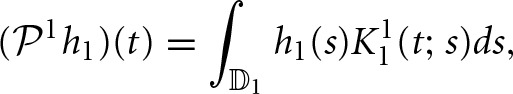



*is called the projection operator from* 𝕃^1^(𝔻_1_) *to* 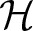
^1^_1_. *Similarly, let h*_2_(*t*_1_, *t*_2_) ∈ 𝕃^1^(𝔻_2_), *the operator* 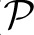
^1^: 𝕃^1^(𝔻_2_) → 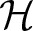
^1^_2_ *(by abuse of notation) given by*

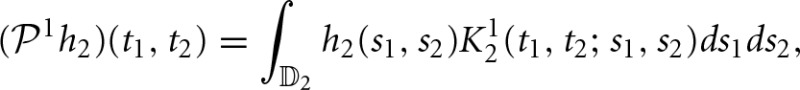



*is called the projection operator from* 𝕃^1^(𝔻_2_) *to* 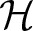
^1^_2_.

#### 2.2.2. Modeling the space of spikes

The feedback kernels of the neural circuit in Figure 1 receive as inputs spike trains generated by the BSGs. Spike trains are often modeled as sequences of Dirac delta pulses and, consequently, the outputs of linear feedback kernels are the result of superposition of their impulse responses (Keat et al., 2001; Pillow et al., 2008; Lazar et al., 2010).

Dirac delta pulses have infinite bandwidth. Spikes generated by the BSGs, however, have limited effective bandwidth. Following (Lazar and Slutskiy, 2014) spikes are modeled to be the RK of an one-dimensional Hilbert space 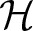
^2^_1_ at spike time occurrence. Here 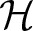
^2^_1_ is a space of trigonometric polynomials whose order *L*^2^, period *S*^2^ and bandwidth Ω^2^ may differ from the input stimulus space 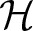
^1^_1_, where Ω^2^ shall be larger than the bandwidth assumed for the feedback kernel, and *S*^2^ is much larger than the support of the feedback kernel (Lazar and Slutskiy, 2014). A spike at time *t*^*i*^_*k*_ of neuron *i* can then be expressed in functional form as *K*^2^_1_(*t*^*i*^_*k*_; *t*), where the superscript indicates that the RK belongs to the spike input space.

Due to the reproducing property, single or pairs of input spikes have the property

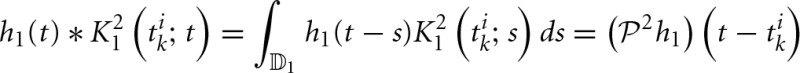



and

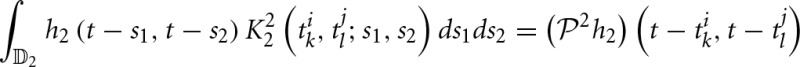



for *i*, *j* = 1, 2, *i* ≠ *j*. The operator 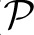
^2^ is similarly defined to 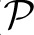
^1^ above; it denotes, however, the projection onto the space of spikes. Thus, not surprisingly, incoming spikes directly readout the projection of the feedback kernels. By letting *L*^2^ → ∞, (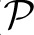
^2^*h*_1_)(*t* − *t*_*k*_) shall converge to *h*_1_(*t* − *t*_*k*_) in 𝕃^2^ norm as the RK converges to the *sinc* function and the RKHS becomes the space of band-limited signals (Lazar et al., 2010). A more detailed analysis is available in Lazar and Slutskiy (2014). This formalism will be employed for solving the functional identification problem formulated in Section 4.1.

### 2.3. Volterra dendritic stimulus processors

As mentioned in Section 2.1, two forms of dendritic stimulus processing appear in our model.

#### 2.3.1. Feedforward Volterra dendritic stimulus processors

The feedforward DSPs are modeled as up to second order terms in the Volterra series. The feedforward DSPs take continuous signals in the stimulus space as inputs, while the output can be expressed as (see also Figure 1)

(9) $$\int_{{𝔻}_{1}}h_{1}^{11i}(t-s)u_{1}(s)ds+\int_{{𝔻}_{2}}h_{2}^{11i}\left(t-s_{1},t-s_{2}\right)u_{1}(s_{1})u_{1}(s_{2})ds_{1}ds_{2},\;$$

where *h*^11*i*^_1_ ∈ 𝕃^1^(𝔻_1_) and *h*^11*i*^_2_ ∈ 𝕃^1^(𝔻_2_) denote, respectively, the first and second order Volterra kernels, *i* = 1, 2. They are assumed to be real, causal and bounded-input bounded-output (BIBO)-stable. It is also assumed that both *h*^11*i*^_1_ and *h*^11*i*^_2_ have finite memory. In addition, *h*^11*i*^_2_ is assumed, without loss of generality, to be symmetric, i.e., *h*^11*i*^_2_ (*t*_1_, *t*_2_) = *h*^11*i*^_2_ (*t*_2_, *t*_1_).

**Example 2.5**. *We present here a Volterra DSP that is akin to a model of dendritic stimulus processing of complex cells in the primary visual cortex (V1). The difference is that the complex cells operate spatio-temporally, whereas in the example given below they operate temporally. We first consider two first order kernels based on Gabor functions*,

$$\begin{array}{l} g_{c}(t)=\exp\left(-\frac{{\left(t-0.13\right)}^{2}}{2\cdot 0.0005}\right)\cos\text{​}\left(2\pi \cdot 10\cdot (t-0.13)\right),\\ g_{s}(t)=\exp\left(-\frac{{\left(t-0.13\right)}^{2}}{2\cdot 0.0005}\right)\sin\text{​}\left(2\pi \cdot 10\cdot (t-0.13)\right). \end{array}$$

**Figure 2:**
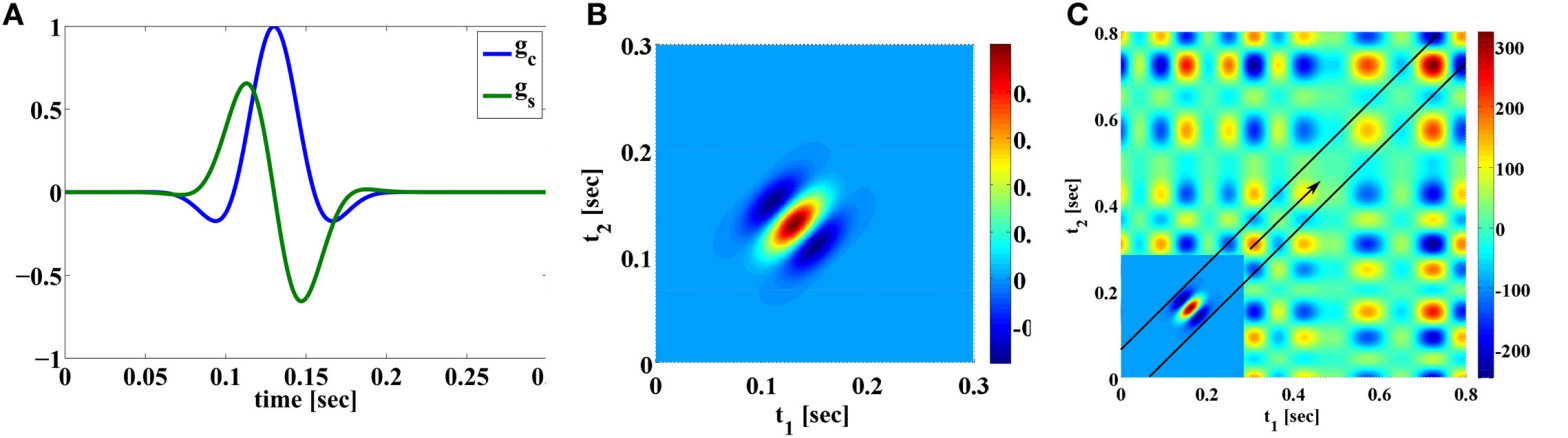
**Examples of Volterra kernels. (A)** First order kernels of quadrature pair of Gabor functions modeling the receptive fields of simple cells. **(B)** Second order kernel modeling receptive fields of complex cells. **(C)** The mechanics of the two dimensional convolution operation between the *u*_2_ (*S*^1^ = 0.8, 𝔻_2_ = [0, 0.8] × [0, 0.8]) and *h*^11*i*^_2_. *u*_2_(*t*_1_, *t*_2_) = *u*_1_(*t*_1_)*u*_1_(*t*_2_) is shown in the background. The inset shows the second order Volterra kernel *h*^11*i*^_2_ rotated 180° around origin [see also **(B)**]. (*h*^11*i*^_2_ is only shown in a restricted domain and is zero elsewhere). For *t* = 0.3, the output of the convolution is the integral of the product of the rotated Volterra kernel and the signal underneath. Since the convolution is evaluated on the diagonal *t* = *t*_1_ = *t*_2_, the second order kernel shifts, as *t* increases, along the arrow on the diagonal. See also Supplementary Figure 5E.

*The two filters are Gaussian modulated sinusoids, that are typically used to model receptive fields of simple cells in the primary visual cortex (V1) where the variables denote space instead of time (Lee, 1996; Dayan and Abbott, 2001). In addition, the two filters are quadrature pair in phase. Both filters are illustrated in Figure 2A. The response of applying the input stimulus u*_1_ *on the temporal filters with impulse response g*_*c*_ *and g*_*s*_ *is given by* ∫_𝔻_1__ *g*_*c*_(*t* − *s*)*u*_1_(*s*)*ds and* ∫_𝔻_1__ *g*_*s*_(*t* − *s*)*u*_1_(*s*)*ds*, *respectively*.

*The responses of the two linear filters of the complex cell model are squared and summed to produce the phase invariant measure v*^*i*^ *(Carandini et al., 2005), where*

(10) $$\begin{array}{l} v^{i}(t)={\left[\int_{{𝔻}_{1}}g_{c}(t-s)u_{1}(s)ds\right]}^{2}+{\left[\int_{{𝔻}_{1}}g_{s}(t-s)u_{1}(s)ds\right]}^{2}\\ \;=\int_{{𝔻}_{2}}g_{c}(t-s_{1})h_{1}(t-s_{2})u_{1}(s_{1})u_{1}(s_{2})ds_{1}ds_{2}\\ \;+\int_{{𝔻}_{2}}g_{s}(t-s_{1})g_{s}(t-s_{2})u_{1}(s_{1})u_{1}(s_{2})ds_{1}ds_{2}\\ \;=\int_{{𝔻}_{2}}\left[g_{c}(t-s_{1})g_{c}(t-s_{2})+g_{s}(t-s_{1})g_{s}(t-s_{2})\right]\\ \;u_{1}(s_{1})u_{1}(s_{2})ds_{1}ds_{2}\\ \;=\int_{{𝔻}_{2}}h_{2}^{11i}(t-s_{1},t-s_{2})u_{1}(s_{1})u_{1}(s_{2})ds_{1}ds_{2}, \end{array}$$

*where h*^11*i*^_2_(*t*_1_, *t*_2_) = *g*_*c*_(*t*_1_)*g*_*c*_(*t*_2_) + *g*_*s*_(*t*_1_)*g*_*s*_(*t*_2_). *Therefore, the operation performed by a complex cell can be modeled with a second order Volterra kernel. *h*^11*i*^_2_ is shown in Figure 2B*.

*We now take a closer look at the operation of the second order kernel. The two dimensional convolution of the second order kernel with u*_2_(*t*_1_, *t*_2_) *is shown in Figure 2C*.

*It is important to note that, since the second order kernel has finite memory, it may not have enough support to cover the entire domain* 𝔻_2_ *for u*_2_(*t*_1_, *t*_2_). *For example, as illustrated in Figure 2C, the output of the second order feedforward DSP at time t is given by the integral of the product of u*_2_(*t*_1_, *t*_2_) *and a rotated h*^11*i*^_2_ *with the origin shifted to* (*t*, *t*) *[see also* (10)*]. Since the shift is along the diagonal, only u*_2_(*t*_1_, *t*_2_) *in the domain that is contained within the black lines is multipled by nonzero values of h*^11*i*^_2_. *u*_2_(*t*_1_, *t*_2_) *elsewhere in the domain is always multiplied by zero in evaluating the output. Therefore, the output of the second order filter only contains information about *u*_2_ within the domain located in between the black lines in Figure 2C. This has implications on decoding the signal (see also Remark 3.11 in Section 3.2)*

#### 2.3.2. Feedback Volterra dendritic stimulus processors

As already mentioned, the feedback DSPs do not operate on stimuli directly but rather on spikes generated by BSGs. We assume that *h*^2*ji*^_1_ ∈ 𝕃^1^(𝔻_1_), *h*^2*ji*^_2_ ∈ 𝕃^1^(𝔻_2_), *i* ≠ *j*, are real, causal, BIBO-stable and have finite memory. In addition, we assume that these kernels are effectively band-limited (see also Section 2.2.2). In functional form we denote a train of spikes as ∑_*k*_ *K*^2^_1_(*t*^*i*^_*k*_; *t*). The output of the feedback DSP *i* amounts to

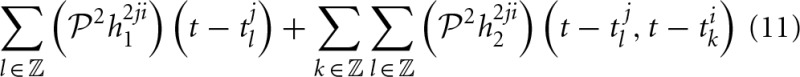



with *j* ≠ *i*.

In particular, the inputs to the second order term of the feedback DSPs are generated by two neurons. This allows for modeling nonlinear interactions between the two neurons in the spike domain.

#### 2.3.3. Overall output from DSPs

The overall inputs (without noise) to the two BSGs in Figure 1 are

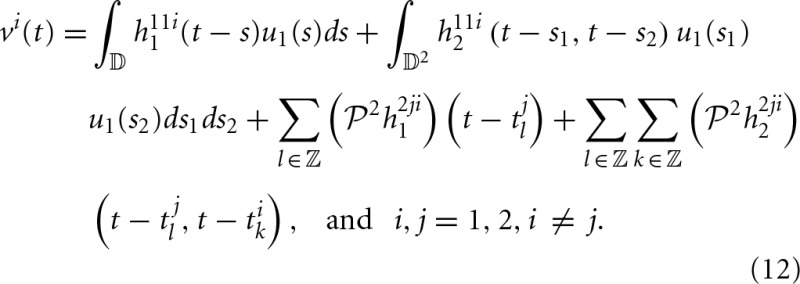



The system of Equations (12) above functionally describe the post-synaptic aggregate currents that are injected into the BSG *i*.

There are a variety of noise sources to be considered. Synaptic variability of feedforward DSPs adds noise sources to the current input to the BSGs. These include thermal noise, synaptic background noise, etc. (Jonston, 1927; Calvin and Stevens, 1968; Manwani and Koch, 1999; Fellous et al., 2003; Destexhe and Rudolph-Lilith, 2012). Feedback DSP kernels may themselves be subject to intrinsic noise sources that may lead to variability in the spike generation process. Intrinsic variability of BSG spike times can, e.g., contribute to the variability of the aggregate current driving the axon hillock in feedback loops.

Overall, the combined effect of DSP noise sources is modeled as Gaussian white noise processes that are added to the feedforward and feedback DSP outputs. The sum total of signal and noise represents the aggregate current input to the BSGs (see Figure 1). Formal DSP noise models will be incorporated directly into the BSG model presented in the next section.

### 2.4. Biophysical spike generators

#### 2.4.1. BSGs and phase response curves

We consider biophysically realistic spike generators such as the Hodgkin-Huxley, Morris-Lecar, Connor-Stevens neurons (Hodgkin and Huxley, 1952; Connor and Stevens, 1971; Morris and Lecar, 1981). The class of BSGs can be expressed in vector notation as

(13) $$\frac{dx^{i}}{dt}=f^{i}\left(x^{i},I^{i}\right),i=1,2,$$

where **x**^*i*^ are the state variables, **f**^*i*^ are vector functions of the same dimension, and *I*^*i*^ are the constant bias currents in the voltage equation of each BSG.

Each input current *v*^*i*^(*t*) is applied to the neuron *i* by additive coupling to the voltage equation, typically the first of the set of ordinary differential equations, i.e.,

(14) $$\frac{dx^{i}}{dt}=f^{i}\left(x^{i},I^{i}\right)+{\left[v^{i}(t),0\right]}^{T},i=1,2,$$

where **0** is a row vector of appropriate size.

We assume that the neuron is periodically spiking when no external input is applied. This can be satisfied by a constant bias current *I*^*i*^ additively coupled onto the voltage equation. The use of *I*^*i*^ is necessary to formulate the encoding for the single neuron case, and this assumption will be relaxed later in this article.

A large enough bias current induces a periodic oscillation of the biophysical spike generator. Therefore, the phase response curve (PRC) is well defined for this limit cycle (Izhikevich, 2007). We denote the PRC of the limit cycle induced by the bias current *I*^*i*^ as $\psi^{i}\left(t,I^{i}\right)={\left[\psi_{1}^{i}\left(t,I^{i}\right),\psi_{2}^{i}\left(t,I^{i}\right),\cdots ,\psi_{N^{i}}^{i}\left(t,I^{i}\right)\right]}^{T}$ with appropriate dimension *N*^*i*^, where ψ^*i*^_*n*_(*t*, *I*^*i*^), *n* = 1, 2, …, *N*^*i*^, are the PRCs associated with the *n*th state variable. Without loss of generality, we assume that ψ^*i*^_1_(*t*, *I*^*i*^) is always the PRC associated with the voltage variable.

An example of a Hodgkin-Huxley neuron model of a BSG can be found in Section 2.2 in the Supplementary Material.

#### 2.4.2. Channel noise in BSGs

As shown in Figure 1, we consider BSGs with noise sources in the ion channels. The noise arises due to thermal fluctuations (White et al., 2000; Hille, 2001) as the finite number of ion channels in the BSGs open and close stochastically.

The differential equations that govern the dynamics of the BSGs in (14) are deterministic. The set of stochastic differential equations (SDEs) below represent their stochastic counterpart (Lazar, 2010):

(15) $$dY^{i}=f^{i}\left(Y^{i},I^{i}\right)dt+B^{i}\left(Y^{i}\right)dZ^{i}(t),i=1,2,$$

where **B**^*i*^ is a matrix with state dependent values, $dZ^{i}={\left[v^{i}dt,dW_{2}^{i},dW_{3}^{i},\cdots ,dW_{P^{i}}^{i}\right]}^{T}$, and *W*^*i*^_*p*_(*t*), *p* = 2, …, *P*^*i*^, are independent Brownian motion processes. Note that *P*^*i*^ does not necessarily have to be equal to *N*^*i*^, the number of state variables. The first element in the stochastic differential *d***Z**^*i*^ is the aggregate dendritic input *v*^*i*^*dt* driving the voltage equation. The other entries in *d***Z**^*i*^ are noise terms that reflect the stochastic fluctuation in the ion channels / gating variables.

Randomness is often added to BSGs by setting **B**^*i*^ = **I**, where **I** is a *N*^*i*^ × *N*^*i*^ identity matrix. The later setting can be viewed as adding subunit noise (Goldwyn and Shea-Brown, 2011). Recently, it has been suggested that a different way of adding channel noise into the BSGs may result in more accurate stochastic behavior (Goldwyn and Shea-Brown, 2011; Goldwyn et al., 2011; Linaro et al., 2011; Orio and Soudry, 2012). The SDEs in (15) are of general form and do not preclude them. In fact, by setting **B**^*i*^ to be a block matrix with blocks equal to be the square root of the diffusion matrix for each ion channel, the channel SDE model (Goldwyn et al., 2011; Orio and Soudry, 2012) can easily be incorporated into (15).

Finally, we note that, under appropriate technical conditions the SDE formulation applies to BSGs with voltage-gated ion channels as well as other types of ion channels. The conditions require that the BSG model can be treated mathematically as a system of SDEs of the form (15) and that the latter satisfies the assumptions of Section 2.4.1.

#### 2.4.3. Overall encoding of the neural circuit model

Taking into account the dendritic input from the feedforward DSPs and feedback DSPs, the encoding by the neural circuit model under the two noise sources is given by two systems of SDEs. With the Brownian motion *W*^*i*^_1_ modeling the DSP white noise, the encoding of neuron *i*, *i* = 1, 2, can be expressed as

(16) $$dY^{i}=f^{i}\left(Y^{i},I^{i}\right)dt+B^{i}\left(Y^{i}\right)dZ^{i}(t),$$

where

$$dZ^{i}=\left[\begin{array}{c} v^{i}dt+dW_{1}^{i}\\ dW_{2}^{i}\\ \vdots \\ dW_{P^{i}}^{i} \end{array}\right],$$

with *v*^*i*^(*t*) given by Equation (12).

Note that in the system of Equations (16) the two output spikes trains (*t*^*i*^_*k*_), *i* = 1, 2, *k* ∈ ℤ, are the observables. Due to the intrinsic noise sources in the DSPs and in the BSGs, spike timing jitter may be observed from trial to trial by repeatedly applying the same stimulus to the neural circuit (see Section 2.3 in the Supplementary Material).

## 3. Encoding, decoding, and noise

In Section 3.1 we present the mathematical encoding formalism underlying the neural circuit in Figure 1. We formulate stimulus decoding as a smoothing spline optimization problem and derive an algorithm that reconstructs the encoded signal in Section 3.2. Finally, we analyze the effect of noise on stimulus decoding in Section 3.3.

### 3.1. Encoding

In this section, we formulate a rigorous stimulus encoding model based on the neural circuit shown in Figure 1. The input of the circuit is a signal *u*_1_ modeling a typical sensory stimulus as described in Section 2.2.1. The neural circuit generates a multidimensional spike train that is assumed to be observable. We establish model equations by first describing the I/O relationship (i.e., the t-transform) of a single BSG. We then provide the *t*-transform of the entire neural circuit model that maps the input stimulus amplitude into a multidimensional spike timing sequence.

#### 3.1.1. The I/O of the BSG

In the presence of a bias current *I*^*i*^ and absence of external inputs, each BSG in Figure 1 is assumed to be periodically spiking. Provided that the inputs are small enough, and by using the PRC, the BSG dynamics of spike generation can be described in an one-dimensional phase space (Lazar, 2010).

**Definition 3.1**. *A neuron whose spike times* (*t*^*i*^_*k*_), *k* ∈ ℤ, *i* = 1, 2, *verify the system of equations*

(17) $$\begin{array}{l} \int_{t_{k}^{i}}^{t_{k+1}^{i}}{\left[\psi^{i}\left(s-t_{k}^{i}+\tau^{i}\left(s-t_{k}^{i},I^{i}\right),I^{i}\right)\right]}^{T}\\ B^{i}\left(x^{i}\left(s-t_{k}^{i}+\tau^{i}\left(s-t_{k}^{i},I^{i}\right),I^{i}\right)\right)dZ^{i}(s)\\ =T^{i}\left(I^{i}\right)-\left(t_{k+1}^{i}-t_{k}^{i}\right), \end{array}$$

where

(18) $$\begin{array}{l} d\tau^{i}\left(t-t_{k}^{i},I^{i}\right)={\left[\psi^{i}\left(t-t_{k}^{i}+\tau^{i}\left(t-t_{k}^{i},I^{i}\right),I^{i}\right)\right]}^{T}\\ \;B^{i}\left(x^{i}\left(t-t_{k}^{i}+\tau^{i}\left(t-t_{k}^{i},I^{i}\right),I^{i}\right)\right)dZ^{i}(t), \end{array}$$

*with* τ^*i*^(0, *I*^*i*^) = 0 *and* **x**^*i*^(*t*, *I*^*i*^) *the periodic solution to* (13) *with bias current *I*^*i*^, is called a Project-Integrate-and-Fire (PIF) neuron with random thresholds. In* (17), [·]^*T*^ *denotes transpose and T*^*i*^(*I*^*i*^) *is the period of limit cycle with bias current *I*^*i*^*.

As its name suggests, the PIF projects a weighted version of the input embedded in noise and the ion channel noise associated with the gating variables (**B**^*i*^*d***Z**^*i*^) onto the PRCs of the corresponding gating variables on a time interval between two consecutive spikes. Note that the integrand in (17) is identical to the RHS of (19). τ^*i*^(*t*, *I*^*i*^) on the LHS of (19) denotes the phase deviation and is driven by the perturbation on the RHS. The LHS of (17) represents the phase deviation measurement performed by the PIF neuron. The RHS of (17) provides the value of the measurement and is equal to the difference between the inter-spike interval and the period of the limit cycle.

The BSG and the PIF neuron with random thresholds are, to the first order, I/O equivalent (Lazar, 2010). In Lazar (2010) it was also shown that a good approximation to the PIF neuron is the reduced PIF with random threshold. The functional description of the reduced PIF is obtained by setting the phase deviation in (17) to zero.

**Definition 3.2**. *The reduced PIF neuron with random threshold is given by the equations*

(19) $$\begin{array}{l} \sum_{n=1}^{N}\int_{t_{k}^{i}}^{t_{k+1}^{i}}\psi_{n}^{i}\left(s-t_{k}^{i},I^{i}\right)b_{n1}^{i}\left(x^{i}\left(s-t_{k}^{i},I^{i}\right)\right)v^{i}(s)ds\\ =T^{i}(I^{i})-\left(t_{k+1}^{i}-t_{k}^{i}\right)+\varepsilon_{k}^{i}, \end{array}$$

*where* (ε^*i*^_*k*_), *k* ∈ ℤ, *is a sequence of independent Gaussian random variables with zero mean and variance*

(20) $$\begin{array}{l} \left(𝔼{\left[\varepsilon_{k}^{i}\right]}^{2}\right)\left(I^{i}\right)\\ \;=\sum_{p=1}^{P^{i}}\int_{t_{k}^{i}}^{t_{k+1}^{i}}{\left[\sum_{n=1}^{N^{i}}\psi_{n}^{i}\left(s-t_{k}^{i},I^{i}\right)b_{np}^{i}\left(x^{i}\left(s-t_{k}^{i},I^{i}\right)\right)\right]}^{2}\text{​​}ds.\; \end{array}$$

For reasons of notational simplicity and without loss of generality, and unless otherwise stated, we shall assume here that **B** = **I** (*N*^*i*^ = *P*^*i*^). The reduced PIF (rPIF) with random threshold can now be written as

(21) $$\int_{t_{k}^{i}}^{t_{k+1}^{i}}\psi_{1}^{i}\left(s-t_{k}^{i},I^{i}\right)v^{i}(s)ds=T^{i}\left(I^{i}\right)-\left(t_{k+1}^{i}-t_{k}^{i}\right)+\varepsilon_{k}^{i},$$

where (ε^*i*^_*k*_), *k* ∈ ℤ, *i* = 1, 2, is a sequence of independent Gaussian random variables with zero mean and variance

(22) $$\left(𝔼{\left[\varepsilon_{k}^{i}\right]}^{2}\right)(I^{i})=\sum_{n=1}^{N^{i}}\int_{t_{k}^{i}}^{t_{k+1}^{i}}{\left[\psi_{n}^{i}(s-t_{k}^{i},I^{i})\right]}^{2}ds.$$

The above analysis assumes that the inputs are weak and therefore the BSGs operate on a limit cycle. Stronger signals can be taken into account by considering a manifold of PRCs associated with a wide range of limit cycles (Kim and Lazar, 2012). By estimating the limit cycle and hence its PRC using spike times, we have the following I/O relationship for each of the BSGs.

**Definition 3.3**. *The reduced PIF neuron with conditional PRC and random threshold is given by the system of equations*

(23) $$\int_{t_{k}^{i}}^{t_{k+1}^{i}}\psi_{1}^{i}\left(s-t_{k}^{i},b_{k}^{i}\right)\left(v^{i}(s)-b_{k}^{i}+I_{0}^{i}\right)ds=\varepsilon_{k}^{i},$$

*where b*^*i*^_*k*_ = [*T*^*i*^]^−1^ (*t*^*i*^_*k* + 1_ − *t*^*i*^_*k*_), *k* ∈ ℤ, *is the total estimated bias current on the inter-spike interval* [*t*^*i*^_*k*_, *t*^*i*^_*k* + 1_], *I*^*i*^_0_ *is an initial bias that brings the neuron close to the spiking region in the absence of input and (by abuse of notation)* ε^*i*^_*k*_, *k* ∈ ℤ, *i* = 1, 2, *is a sequence of independent Gaussian random variables with zero mean and variance*

(24) $$\left(𝔼{\left[\varepsilon_{k}^{i}\right]}^{2}\right)\left(b_{k}^{i}\right)=\sum_{n=1}^{N^{i}}\int_{t_{k}^{i}}^{t_{k+1}^{i}}{\left[\psi_{n}^{i}\left(s-t_{k}^{i},b_{k}^{i}\right)\right]}^{2}ds,$$

*and* ψ^*i*^_1_(*s*, *b*^*i*^_*k*_) *is the conditional PRC (Kim and Lazar, 2012)*.

The conditional PRC formulation above allows us to separate BSG inputs into a constant bias current and fluctuations around it on short inter-spike time intervals. The bias current can be estimated between consecutive spikes, making the deviation from the limit cycle small in each inter-spike interval even for strong inputs. Moreover, by considering the conditional PRCs, the assumption that BSGs oscillate in the absence of input can be nearly dropped. Thus, it is not required for BSGs to always be on a limit cycle. Only when the neuron enters the limit cycle do we consider formulating the encoding using the rPIF model with conditional PRCs.

**Remark 3.4**. *Note that by parametrizing each of the PRCs with *b*^*i*^_*k*_, the variance of the error in* (24) *depends on the estimated PRC on each inter-spike interval. In conjunction with* (23), *we see that the variability of spike times depends on the strength of the input to the BSGs*.

#### 3.1.2. The t-transform of the neural circuit

The overall encoding by the neural circuit model can be expressed as

$$\begin{array}{l} \int_{t_{k}^{i}}^{t_{k+1}^{i}}\psi_{1}^{i}\left(s-t_{k}^{i},b_{k}^{i}\right)v^{i}(s)ds\\ =\left(b_{k}^{i}-I^{i}\right)\int_{t_{k}^{i}}^{t_{k+1}^{i}}\psi_{1}^{i}\left(s-t_{k}^{i},b_{k}^{i}\right)ds+\varepsilon_{k}^{i},i=1,2,k\in \mathbb{Z}. \end{array}$$

Substituting (12) into the above, we have

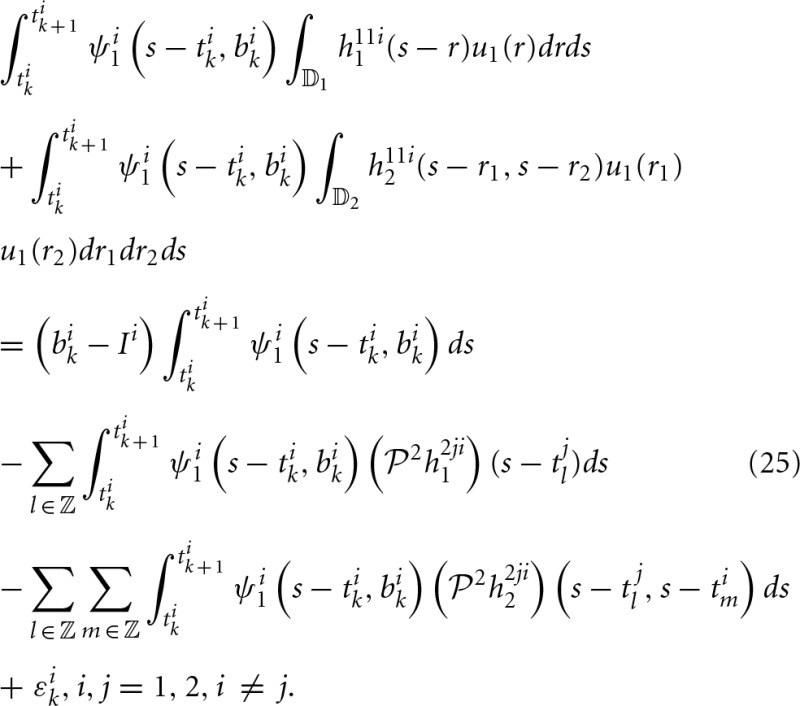



We arrived at the following.

**Lemma 3.5**. *The model of encoding in Figure 1 is given in operator form by*

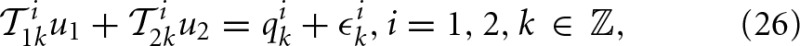



*where u*_1_ ∈ 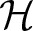
^1^_1_, *u*_2_ ∈ 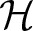
^1^_2_, *u*_2_(*t*_1_, *t*_2_) = *u*_1_(*t*_1_)*u*_1_(*t*_2_), *and*, 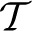
^*i*^_1*k*_: 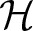
^1^_1_ → ℝ *and* 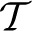
^*i*^_2*k*_: 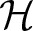
^1^_2_ → ℝ *are bounded linear functionals given by*

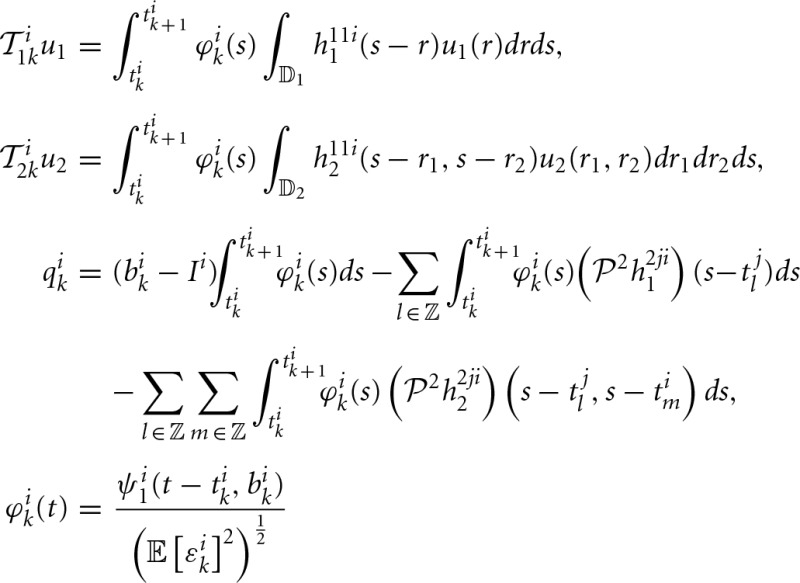



*and* ϵ^*i*^_*k*_, *k* ∈ ℤ, *are independent random variables with normal distribution* 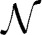
(0, 1) *and j* = 1, 2, *j* ≠ *i*. *Equation (26) is called the t-transform (Lazar and Tóth, 2004) of the neural circuit in Figure 1*.

**Remark 3.6**. *The t-transform describes the mapping of the input stimulus *u*_1_ into the spike timing sequence* (*t*^*i*^_*k*_), *i* = 1, 2, *k* ∈ ℤ. *Thus, the t-transform shows how the amplitude information of the input signal is related to or transformed into the time information contained in the sequence of output spikes generated by the neural circuit*.

We provide here further intuition behind the Equations (26). By the Riesz representation theorem (Berlinet and Thomas-Agnan, 2004), there exists functions ϕ^*i*^_1*k*_ ∈ 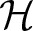
^1^_1_ such that

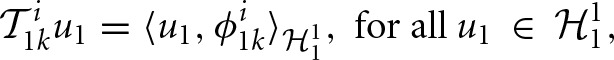



and ϕ^*i*^_2*k*_ ∈ 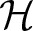
^1^_2_ such that

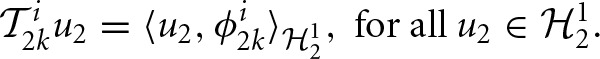



Therefore, (26) can be rewritten in inner product form:

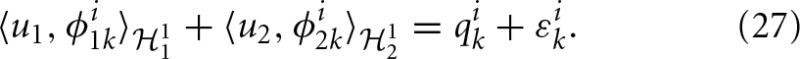



Recall that inner products are projections that are typically interpreted as measurements. In the Equation (27) above, the signals *u*_1_ and *u*_2_ are projected onto the sampling functions ϕ^*i*^_1*k*_ and ϕ^*i*^_2*k*_, respectively. We also note that traditional amplitude sampling of a bandlimited signal *u*_1_ at times (*t*_*n*_), *n* ∈ ℤ, can be expressed as

$${\langle u_{1}(\cdot ),\mathrm{sinc}(t_{n}-\cdot )\rangle }_{{𝕃}^{2}(\mathbb{R})}=u_{1}(t_{n}),$$

where $\mathrm{sinc}(t)=\frac{\sin(\Omega^{1}t)}{\pi t}$ is the impulse response of the ideal low pass filter bandlimited to Ω^1^ or in other words, the kernel of the RKHS of finite-energy band-limited functions (Lazar and Pnevmatikakis, 2009). Thus, the neural encoding model described by the Equation (27) can be interpreted as generalized sampling with noisy measurements with sampling functions ϕ^*i*^_1*k*_ and ϕ^*i*^_2*k*_.

The formulation of the encoding model can easily be extended to the case when *M* neural circuits encode a stimulus in parallel. This is shown schematically in Supplementary Figure 1. A left superscript was added in the figure to each of the components to indicate the circuit number.

### 3.2. Decoding

In the previous section, we showed that the encoding of a signal *u*_1_ by the neural circuit model with feedforward and feedback DSPs and BSGs can be characterized by the set of t-transform Equations (26). We noticed that the Equations (26) are nonlinear in *u*_1_ due to the second order Volterra term. However, by reinterpreting the second order term as linear functionals 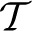
^*i*^_2*k*_ on the higher dimensional tensor space 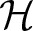
^1^_2_, (26) implies that the measurements taken by each of the neurons are the sum of linear measurements in two different vector spaces [see also Equations (27)].

In this section we investigate the decoding of signals encoded with the neural circuit in Figure 1. The purpose of decoding is to recover from the set of spike times the original signals, *u*_1_(*t*) and *u*_2_(*t*_1_, *t*_2_), that respectively belong to the two different vector spaces 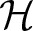
^1^_1_ and 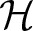
^1^_2_. We formulate the decoding problem as the joint smoothing spline problem

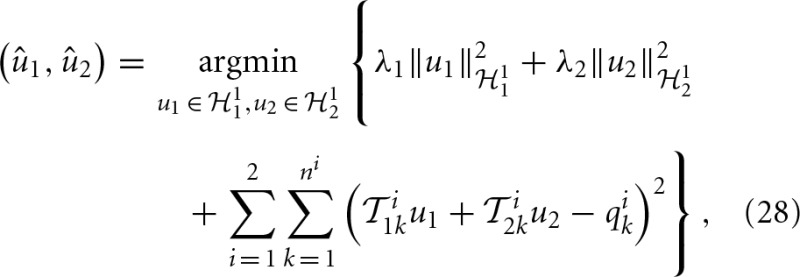



where *n*^*i*^ + 1 is the number of spikes generated by BSG *i* = 1, 2.

**Theorem 3.7**. *The solution to (28) is of the form*

(29) $$\begin{array}{l} \;{\hat{u}}_{1}(t)=\sum_{i=1}^{2}\sum_{k=1}^{n^{i}}c_{k}^{i}\phi_{1k}^{i}(t)\\ {\hat{u}}_{2}(t_{1},t_{2})=\sum_{i=1}^{2}\sum_{k=1}^{n^{i}}c_{k}^{i}\phi_{2k}^{i}(t_{1},t_{2}), \end{array}$$

*where* ϕ^*i*^_1*k*_(*t*) = 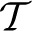
^*i*^_1*k*_*K*^1^_1|*t*_ and ϕ^*i*^_2*k*_(*t*_1_, *t*_2_) = 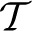
^*i*^_2*k*_*K*^1^_2|*t*_1_, *t*_2__, *i* = 1, 2, *k* = 1, …, *n*^*i*^,

$c={\left[c_{1}^{1},\cdots ,c_{n^{1}}^{1},c_{1}^{2},\cdots ,c_{n^{2}}^{2}\right]}^{T}$ *is the solution of the system of linear equations*

(30) $$\left({\left(\Phi_{1}+\Phi_{2}\right)}^{2}+\lambda_{1}\Phi_{1}+\lambda_{2}\Phi_{2}\right)c=\left(\Phi_{1}+\Phi_{2}\right)q,$$

*where* $q={\left[q_{1}^{1},\cdots ,q_{n^{1}}^{1},q_{1}^{2},\cdots ,q_{n^{2}}^{2}\right]}^{T}$, *and*

$$\Phi_{i}=\left[\begin{array}{c} \begin{array}{l} \Phi_{i}^{11}\Phi_{i}^{12}\\ \Phi_{i}^{21}\Phi_{i}^{22} \end{array} \end{array}\right],i=1,2,$$

and

$${\left[\Phi_{i}^{mn}\right]}_{kl}=\langle \phi_{ik}^{m},\phi_{il}^{n}\rangle .$$

**Proof:** Proof of the theorem follows the Representer Theorem (Berlinet and Thomas-Agnan, 2004) and is given in detail in Appendix.

**Remark 3.8**. *When* λ_1_ = λ_2_, *the solution* **c** *amounts to*

$$c={\left(\Phi_{1}+\Phi_{2}+\lambda_{1}I\right)}^{-1}q,$$

*where* **I** *is an identity matrix of appropriate dimensions*.

**Remark 3.9**. *Although* (29) *solves* (28), *in practice a minimum number of spikes is needed to obtain a meaningful estimate of the original signal. A minimum bound for the number of measurements/spikes can be derived in the noiseless case. Clearly, the bound has to be larger than the dimension of the space. This may require the signal to be encoded by a circuit with a larger number of neurons than the two shown in Figure 1 (Lazar and Slutskiy, in press). A number of such neural circuits in parallel can be used to encode input stimuli as shown in the Supplementary Figure 1. Theorem 3.7 can be easily extended to solving the smoothing spline problem*

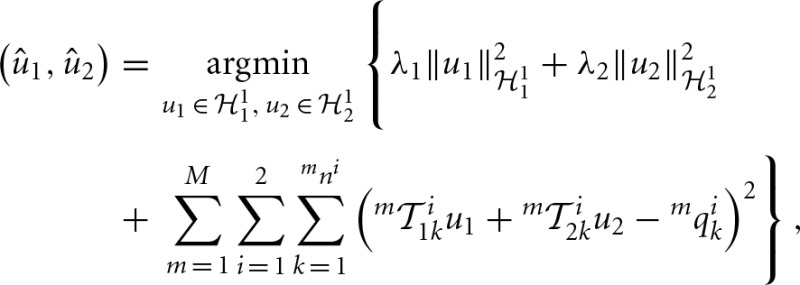



*where *m* = 1, 2, …, *M*, denotes the circuits number in Supplementary Figure 1. In addition, if the circuits consist of only first order feedforward kernels, then only *u*_1_(*t*) can be reconstructed. Similarly, if the circuits are comprised of only the second order feedforward kernels, then *u*_2_(*t*_1_, *t*_2_) can be reconstructed but not *u*_1_(*t*)*.

**Remark 3.10**. *Since u*_2_(*t*_1_, *t*_2_) = *u*_1_(*t*_1_)*u*_1_(*t*_2_) = *u*_2_(*t*_2_, *t*_1_), *u*_2_ *belongs to a subspace of* 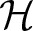
^1^_2_ *whose elements are symmetric functions. We also note that since the second order feedforward kernels are symmetric, the sampling functions* (ϕ^*i*^_2*k*_(*t*_1_, *t*_2_)), *i* = 1, 2, *k* = 1, …, *n*^*i*^, *also belong to the same subspace. Therefore, if the sampling functions span the subspace of symmetric functions in* 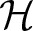
^1^_2_, *u*_2_ *can readily be reconstructed with only* (*L*^1^ + 1)(2*L*^1^ + 1) *measurements/spikes, rather than* (2*L*^1^ + 1)^2^, *the dimension of* 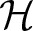
^1^_2_.

**Remark 3.11**. *The reconstruction of u*_2_(*t*_1_, *t*_2_) *on* 𝔻_2_ *strongly depends on the support (in practice the finite memory) of the kernels h*^11*i*^_2_, *i* = 1, 2 *(see also Figure 2C). In the reconstruction example of the Supplementary Figure 5, we show that* ${\hat{u}}_{2}$ *approximates u*_2_ *well in the restricted domain where h*^11*i*^_2_ *is nonzero. Outside this restricted domain, h*^11*i*^_2_ *vanishes and u*_2_ *is not well recovered as suggested by the large error in the Supplementary Figure 5E*.

### 3.3. Effect of noise on stimulus decoding

In this section, we investigate the effect of noise sources (i) on spike timing of the reduced PIF neuron, and (ii) on the decoding of stimuli encoded with a neural circuit. We will also present the effect of an alternative noise source model on both spike timing and stimulus decoding.

#### 3.3.1. Effect of noise on measurement and spike timing errors of the reduced PIF neuron

As suggested by (22), the variance of the measurement error of the reduced PIF neuron is directly related to the PRC of the associated limit cycle. We first characterize the variance of the measurement error due to each individual noise source parametrized by the bias current *I*^*i*^. We then evaluate the spike timing variance between the spike trains generated by the Hodgkin-Huxley neuron and the reduced PIF neuron again as a function of the bias current *I*^*i*^. We start with a brief description of the key elements of Hodgkin-Huxley neuron and the PIF neuron.

We consider the stochastic Hodgkin-Huxley equations

(31) $$dY^{i}=f^{i}\left(Y^{i},I^{i}\right)dt+dZ^{i}(t),$$

where **f**^*i*^ is defined as in Section 2.2 of the Supplementary Material with additional normalization such that the unit of time is in seconds instead of milliseconds and the unit of voltage is in Volts instead of milivolts as conventionally used. **Z**^*i*^(*t*) takes the form

$$dZ^{i}(t)=\left[\begin{array}{c} \begin{array}{l} v^{i}dt+\sigma_{1}^{i}dW_{1}^{i}\\ \;\sigma_{2}^{i}dW_{2}^{i}\\ \;\sigma_{3}^{i}dW_{3}^{i}\\ \;\sigma_{4}^{i}dW_{4}^{i} \end{array} \end{array}\right].$$

Here *W*^*i*^_*n*_(*t*) are independent standard Brownian motion processes and σ^*i*^_*n*_, *n* = 1, 2, 3, 4, are associated scaling factors.

The variance of the measurement error of the reduced PIF neuron due to each Brownian motion process *W*^*i*^_*n*_, *n* = 1, …, 4, is given by [see also Equation (22)]

(32) $$\left(𝔼{\left[\varepsilon_{kn}^{i}\right]}^{2}\right)\left(I^{i}\right)={\left(\sigma_{n}^{i}\right)}^{2}\int_{t_{k}^{i}}^{t_{k+1}^{i}}{\left[\psi_{n}^{i}\left(s-t_{k}^{i},I^{i}\right)\right]}^{2}ds.$$

**Figure 3:**
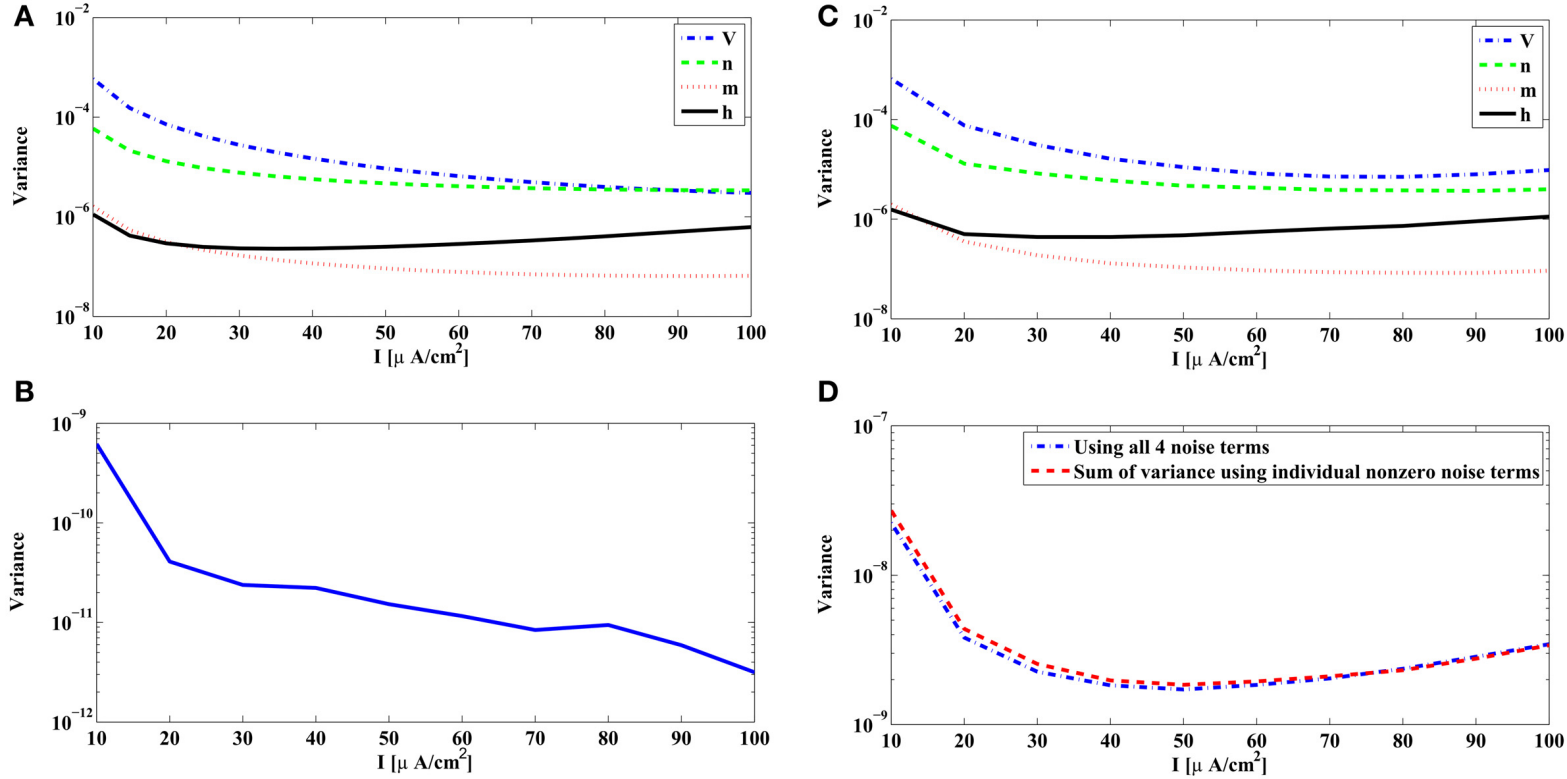
**Variance of the measurement and spike timing errors. (A)** Error measurement variances computed from the PRCs of the Hodgkin-Huxley neuron [Equation (32)]. Each individual variance is parametrized by the bias current *I*^*i*^. **(B)** Error variance between spike times generated by the noiseless Hodgkin-Huxley neuron and its reduced PIF counterpart. (**C)** The spike timing error variance due to each source of noise, obtained from simulations of the Hodgkin-Huxley neuron follow the pattern of the theoretically derived measurement error shown in **(A)**. The spike timing error variances are obtained by setting, at each time, one of the σ_*n*_'s to a nonzero value and the rest to zero. The spikes generated by the Hodgkin-Huxley neuron are compared with the spikes generated by its reduced PIF counterpart. The variance of the differences between two spike times are normalized by the nonzero σ_*n*_ mentioned before. **(D)** The spike timing variance due to the simultaneous presence of multiple noise sources approximates the sum of spike timing variances due to individual noise sources. Blue curve shows the spike timing variance obtained by simulating Hodgkin-Huxley equations using nonzero values for all σ_*n*_, *n* = 1, 2, 3, 4. Red curve shows the sum of spike timing variances obtained in **(C)** with proper scaling.

We show in Figure 3A the variance of the measurement error in (32) associated with each source of noise of the reduced PIF neuron for the unitary noise levels σ^*i*^_*n*_ = 1, *n* = 1, 2, 3, 4. The variances given by (32) are plotted as a function of the bias current *I*^*i*^. Clearly, the noise arising in dendritic stimulus processing (*W*^*i*^_1_) induces the largest error, and together with noise in the potassium channels (*W*^*i*^_2_), these errors are about two magnitudes larger in variance than those induced by the noise sources in the sodium channels (*W*^*i*^_3_, *W*^*i*^_4_).

The above analysis is based on the analytical derivation of the measurement error in (32) for the rPIF neurons. The measurement error is closely related, however, to the spike timing variation of the BSGs subject to noise sources. A variance of 10^−6^ in Figure 3A corresponds to a standard deviation of 1 ms in spike timing. In practice the error between the spike times of the Hodgkin-Huxley neuron and the reduced PIF neuron can be directly evaluated.

In order to do so, we randomly generated a weak bandlimited dendritic input. All evaluations were based on encoding a signal with the Hodgkin-Huxley neuron model described above with internal noise sources and bias current *I*^*i*^. The spike times (*t*^*i*^_*k*_) of the Hodgkin-Huxley neuron were recorded. Starting from each spike time *t*^*i*^_*k*_, we encoded the appropriate portion of the signal by the reduced PIF neuron until a spike ^*r*^*t*^*i*^_*k* + 1_ was generated. The difference between ^*r*^*t*^*i*^_*k* + 1_ and *t*^*i*^_*k* + 1_ is the error in approximating the encoding using the reduced PIF formulation. This process was repeated for each *I*^*i*^. We computed the variance of the errors based on some 3000–5000 spikes generated in encoding the input.

In Figure 3B, the variance of the spike timing error ^*r*^*t*^*i*^_*k* + 1_ − *t*^*i*^_*k* + 1_ for σ_*n*_ = 0, *n* = 1, 2, 3, 4, is shown. Since the reduced PIF is an approximation (even under noiseless conditions) and, although small, the error is nonzero. From Figure 3B, the variance of the spike timing error is on the order of 10^−9^. We shall evaluate the spike timing error variance of the intrinsic noise sources in a range much larger than 10^−9^.

We also tested to what extent each individual source of noise contributes to the variance of spike timing as suggested by the theoretical analysis depicted in Figure 3A. Indeed, the error variance obtained through simulations in Figure 3C follows the basic pattern shown in Figure 3A. Figure 3C was obtained by setting one of the σ_*n*_'s to a nonzero value and the rest to 0 (the nonzero values were σ_1_ = σ_2_ = 0.01, σ_3_ = σ_4_ = 0.1). Each nonzero value was picked to be large enough so that the error variance in the absence of noise (Figure 3B) becomes negligible, and at the same time, it was small enough such that the states of the neurons did not substantially deviate from the limit cycle. To compare the with the ones in Figure 3A we normalized the error variance obtained in simulations by σ_*n*_.

Next, we tested whether the variance of spike timing due to presence of multiple noise sources is truly the summation of error variances due to individual noise sources. We simulated the Hodgkin-Huxley equations with σ_1_ = σ_2_ = 0.005, σ_3_ = σ_4_ = 0.05. The total spike timing error variance shown in Figure 3D (blue curve) is very close to the sum of error variances in Figure 3C with proper scaling (red curve in Figure 3D).

As suggested by the above analysis, the reduced PIF neuron with random thresholds largely captures the encoding of stimuli by BSGs subject to intrinsic noise sources.

#### 3.3.2. Effect of noise on stimulus decoding

In order to quantitatively explore how noise impacts signal decoding, we recovered from spikes the signal encoded by the noisy neural circuit of Supplementary Figure 1. We started with the base-level noise-less case described in Section 3.2 of the Supplementary Material (*M* = 4) and proceeded to introduce individual noise terms with a range of scaling factors. For example, we set σ^*i*^_2_ = σ^*i*^_3_ = σ^*i*^_4_ = 0 and varied σ^*i*^_1_. We also tested the case when 10σ^*i*^_1_ = 10σ^*i*^_2_ = σ^*i*^_3_ = σ^*i*^_4_ for the aggregated effect on stimulus recovery. We choose to use σ^*i*^_3_ and σ^*i*^_4_ 10 times larger than σ^*i*^_1_ and σ^*i*^_2_ so that each noise source introduced a similar error.

In all simulations, the Euler-Maruyama scheme (Kloeden and Platen, 1992) was used for the numerical integration of the SDEs. We performed 20 encoding and decoding experiments. Each time, the input stimulus was generated by randomly picking from a Gaussian distribution the real and imaginary parts of the coefficients *u*_*l*_ in (1). We further constrained the stimuli to be real-valued. (An example is given in Supplementary Figure 5.) For each noise level, the input signal was encoded/decoded. The mean Signal-to-Noise Ratio (SNR) across 20 experiments is reported for each noise level. The SNR for the reconstruction of *u*_1_ was computed as

(33) $$\text{SNR}=10\log_{10}\left[\frac{\|u_{1}{\|}^{2}}{\|u_{1}-{\hat{u}}_{1}{\|}^{2}}\right],$$

where *u*_1_ is the original signal and ${\hat{u}}_{1}$ is its reconstruction. Note that the spike time occurrences generated for the same signal are different for each noise level. Since the sampling functions are spike time dependent, the number of measurements/spikes may not be the same for each noise level. Moreover, at times, the sampling functions may not fully span the stimulus space. To reduce the uncertainty caused by the stimulus dependent sampling we averaged our SNR data over 20 different signals.

**Figure 4:**
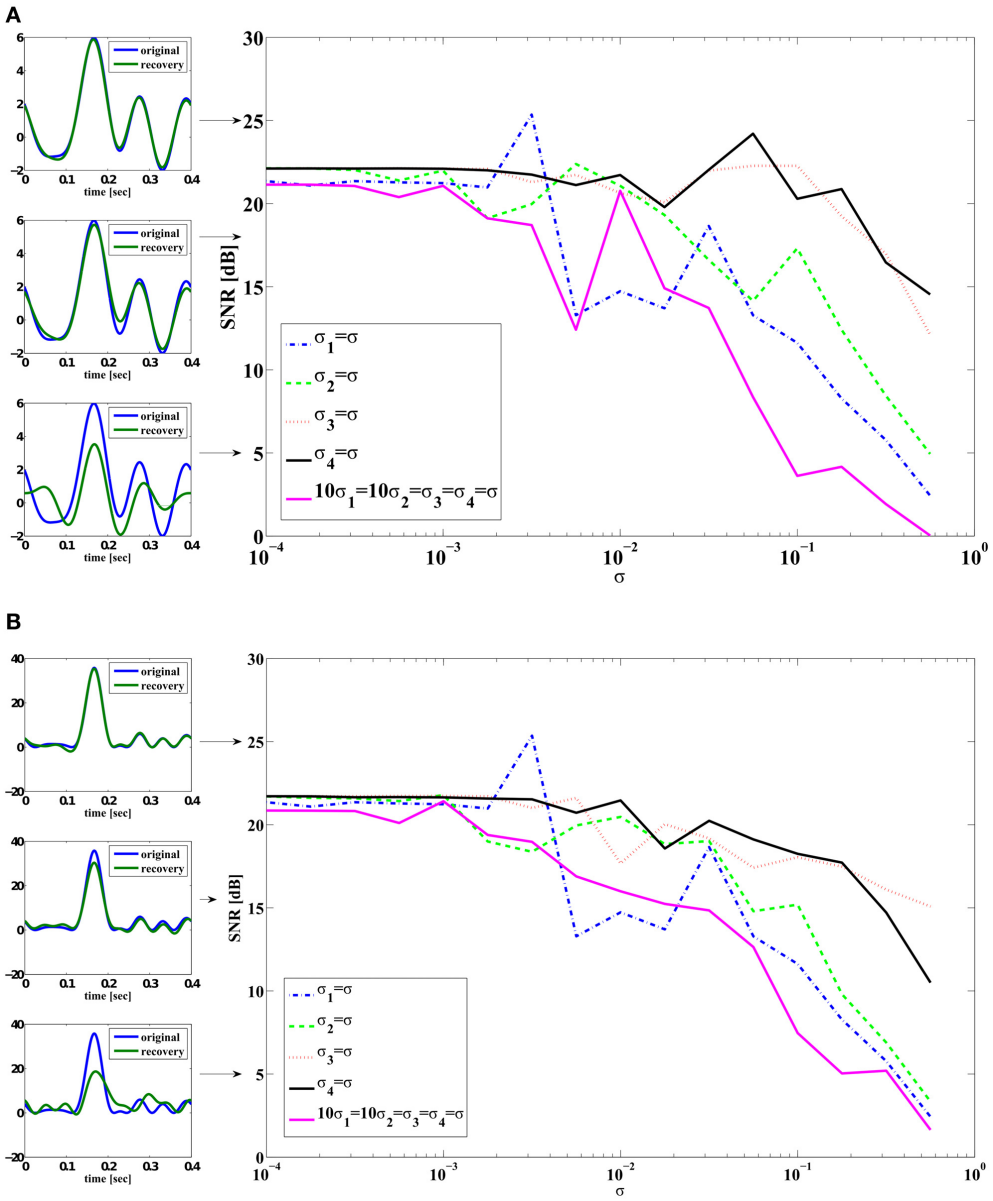
**SNR reconstruction error of encoded signals with a total of *M* = 2 circuits (4 neurons)**. Color legend: (Blue) σ^*i*^_1_ = σ, σ^*i*^_2_ = σ^*i*^_3_ = σ^*i*^_4_ = 0. (Green) σ^*i*^_2_ = σ, σ^*i*^_1_ = σ^*i*^_3_ = σ^*i*^_4_ = 0. (Red) σ^*i*^_3_ = σ, σ^*i*^_1_ = σ^*i*^_2_ = σ^*i*^_4_ = 0. (Black) σ^*i*^_4_ = σ, σ^*i*^_1_ = σ^*i*^_2_ = σ^*i*^_3_ = 0. (Magenta) 10σ^*i*^_1_ = 10σ^*i*^_2_ = σ^*i*^_3_ = σ^*i*^_4_ = σ. In-sets (on the left) are typical reconstructions that yield corresponding SNR indicated by arrows. The top left in **(A)** shows an example of reconstruction (green) whose SNR is 25 dB when compared to the original signal (blue). **(A)** SNR of reconstruction of *u*_1_(*t*). **(B)** SNR of reconstruction of *u*^2^_1_(*t*) = *u*_2_(*t*, *t*).

Figure 4A shows the SNR of the reconstruction of signal *u*_1_(*t*) against different noise strength. Figure 4B shows the SNR of the reconstruction of signal *u*^2^_1_(*t*) = *u*_2_(*t*, *t*). The reconstruction SNR in Figure 4A largely matches the inverse ordering of noise strength of each of the individual noise sources shown in Figure 3A. DSP noise sources degrade the reconstruction performance most strongly while noise sources associated with gating variables *m* and *h* have a much smaller effect for the same variance level. Since the variance of measurement error is the sum of error variance in each variable, the case when 10σ_1_ = 10σ_2_ = σ_3_ = σ_4_ = σ exhibits the lowest performance.

#### 3.3.3. Effect of an alternative noise model on spike timing and stimulus decoding

Biologically, the effect of channel noise on the operation of the BSGs is due to the ON-OFF activity of a finite number of ion channels. The Hodgkin-Huxley equations and the noise terms used in Section 3.3.2 correctly capture the dynamics in the limit of infinitely many channels. Recent research, however, suggests that the model equations may not correctly model the ion current fluctuations for a finite number of channels (Goldwyn and Shea-Brown, 2011).

We consider here an alternative stochastic formulation of the Hodgkin-Huxley model that more precisely captures the ion channel kinetics. By using a finite number of ion channels the strength of noise amplitude becomes directly related to the actual number of ion channels. Therefore, the free variables are only the number of potassium and sodium channels that are both biologically meaningful. The successful use of an alternative noise model as described below also suggests that our analysis can be applied to a wide range of stochastic formulations of BSGs based on SDEs.

We shall construct here stochastic ion channels using conductance noise rather than subunit noise as in the previous Sections (Goldwyn and Shea-Brown, 2011; Goldwyn et al., 2011). This stochastic Hodgkin-Huxley system is simulated using a diffusion approximation following (Orio and Soudry, 2012). The system of SDEs can be expressed as

$$dY^{i}=f^{i}\left(Y^{i},I^{i}\right)dt+B^{i}\left(Y^{i}\right)dZ^{i}(t),$$

where **Y**^*i*^ has 14 state variables and the full system can be found in Section 3.3 of the Supplementary Material. Here *i* = 1 for simplicity.

The variance of the measurement error is now given by (20). We can decompose the variance into three terms as

$$𝔼{\left[\varepsilon_{k}^{i}\right]}^{2}=𝔼{\left[\varepsilon_{kV}^{i}\right]}^{2}+𝔼{\left[\varepsilon_{kK}^{i}\right]}^{2}+𝔼{\left[\varepsilon_{kNa}^{i}\right]}^{2},$$

where ε^*i*^_*kV*_, ε^*i*^_*kK*_, ε^*i*^_*kNa*_ are measurement errors associated with the noise in the DSP, in potassium channels and in sodium channels, respectively.

As ε^*i*^_*kV*_ is quantitatively the same as that in Section 3.3.2, we focus here on ε^*i*^_*kK*_ and ε^*i*^_*kNa*_. The variance of the errors can be respectively expressed as

$$\begin{array}{l} \left(𝔼{\left[\varepsilon_{kK}^{i}\right]}^{2}\right)(I^{i})\\ =\sum_{p=2}^{5}\int_{t_{k}^{i}}^{t_{k+1}^{i}}{\left[\sum_{n=2}^{6}\psi_{n}^{i}(s-t_{k}^{i},I^{i})b_{np}^{i}\left(x^{i}(s-t_{k}^{i},I^{i})\right)\right]}^{2}ds, \end{array}$$

and

$$\begin{array}{l} \left(𝔼{\left[\varepsilon_{kNa}^{i}\right]}^{2}\right)(I^{i})\\ =\sum_{p=6}^{15}\int_{t_{k}^{i}}^{t_{k+1}^{i}}{\left[\sum_{n=7}^{14}\psi_{n}^{i}\left(s-t_{k}^{i},I^{i}\right)b_{np}^{i}\left(x^{i}(s-t_{k}^{i},I^{i})\right)\right]}^{2}ds. \end{array}$$

Note that *b*_*np*_, *n* = 1, …, 14, *p* = 2, 3, …, 15, are functions that dependent on either the number of potassium channels *N*_*Na*_ or the number of sodium channels *N*_*K*_, and the states of the neuron.

**Figure 5:**
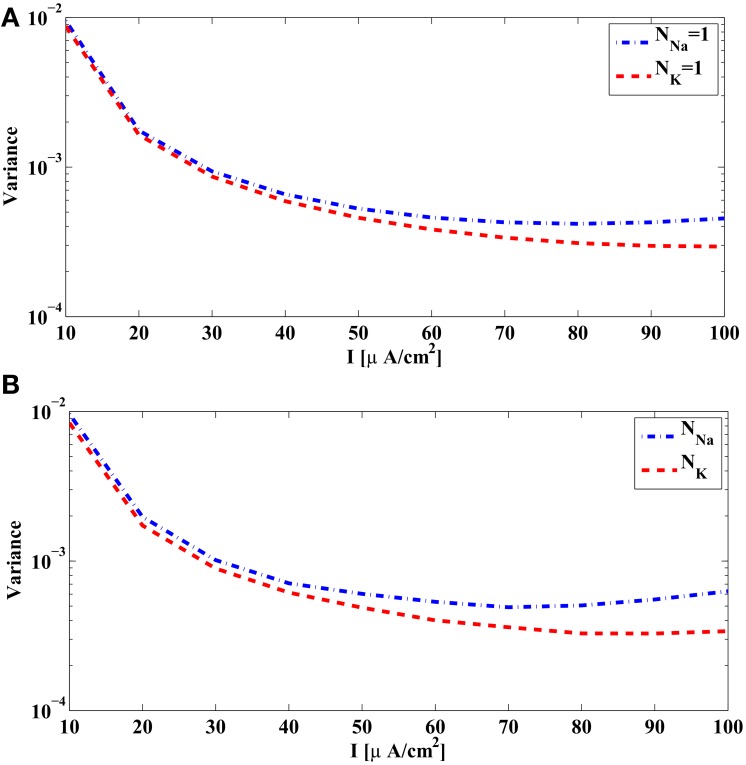
**The variance of the measurement and spike timing error associated with the sodium channels (blue) and the potassium channels (red) of the Hodgkin-Huxley equations with alternative noise sources parametrized by the bias current *I*. (A)** The variance of the measurement error computed from PRCs of Hodgkin-Huxley equations, with *N*_*Na*_ = 1 and *N*_*K*_ = 1. Actual variance with different number of ion channels is inversely proportional to *N*_*Na*_ and *N*_*K*_, respectively. **(B)** Spike timing variance obtained in simulations by comparing the spike times generated by the Hodgkin-Huxley neuron with channel noise and the spike times generated by its reduced PIF counterpart. Blue curve is obtained by using *N*_*Na*_ = 5 × 10^4^, *N*_*K*_ = ∞, and normalized to 1 sodium channel. Red curve is obtained by using *N*_*K*_ = 5 × 10^4^, *N*_*Na*_ = ∞, and normalized to 1 potassium channel.

We first evaluate (𝔼[ε^*i*^_*kNa*_]^2^)(*I*^*i*^) using the PRCs. The PRCs are obtained by letting *N*_*Na*_ = *N*_*K*_ = ∞ and thereby making the system deterministic. Since the measurement error variance for fixed *I*^*i*^ is proportional to (*N*_*Na*_)^−1^, it is shown in Figure 5A as a function of the bias current *I*^*i*^ for *N*_*Na*_ = 1. Similarly, the variance of the measurement error $\left(E{\left[\varepsilon_{kK}^{i}\right]}^{2}\right)(I^{i})$ for *N*_*K*_ = 1 is shown in Figure 5A, and it is proportional to (*N*_*K*_)^−1^ for a fixed *I*^*i*^. We notice that, when the number of channels is the same, the measurement error due to the sodium channels is of the same order of magnitude with the measurement error due to the potassium channels. However, the number of sodium channels is typically 3–4 times larger than the number of potassium channels. Therefore, in contrast to the previous case, the error induced by sodium channels shall be larger than that induced by potassium channels.

We also analyzed in simulations the difference between spike times generated by the alternative stochastic formulation of the Hodgkin-Huxley equations and those generated by the corresponding reduced PIF neuron. We used in simulation *N*_*Na*_ = 5 × 10^4^, *N*_*K*_ = ∞, to obtain the variance $\left(E{\left[\varepsilon_{kNa}^{i}\right]}^{2}\right)(I^{i})$ and scaled it by *N*_*Na*_ to compare it with Figure 5A. Similarly, we used *N*_*K*_ = 5 × 10^4^, *N*_*Na*_ = ∞, to obtain the variance $E{\left[\varepsilon_{kK}^{i}\right]}^{2}(I^{i})$. The spike timing variances of error across different *I*^*i*^ are shown in Figure 5B The pattern of similarity between variances in Figures 5A,B suggest that the reduced PIF with random threshold associated with this formulation of Hodgkin-Huxley equations is highly effective in capturing the encoding under internal noise sources.

**Figure 6:**
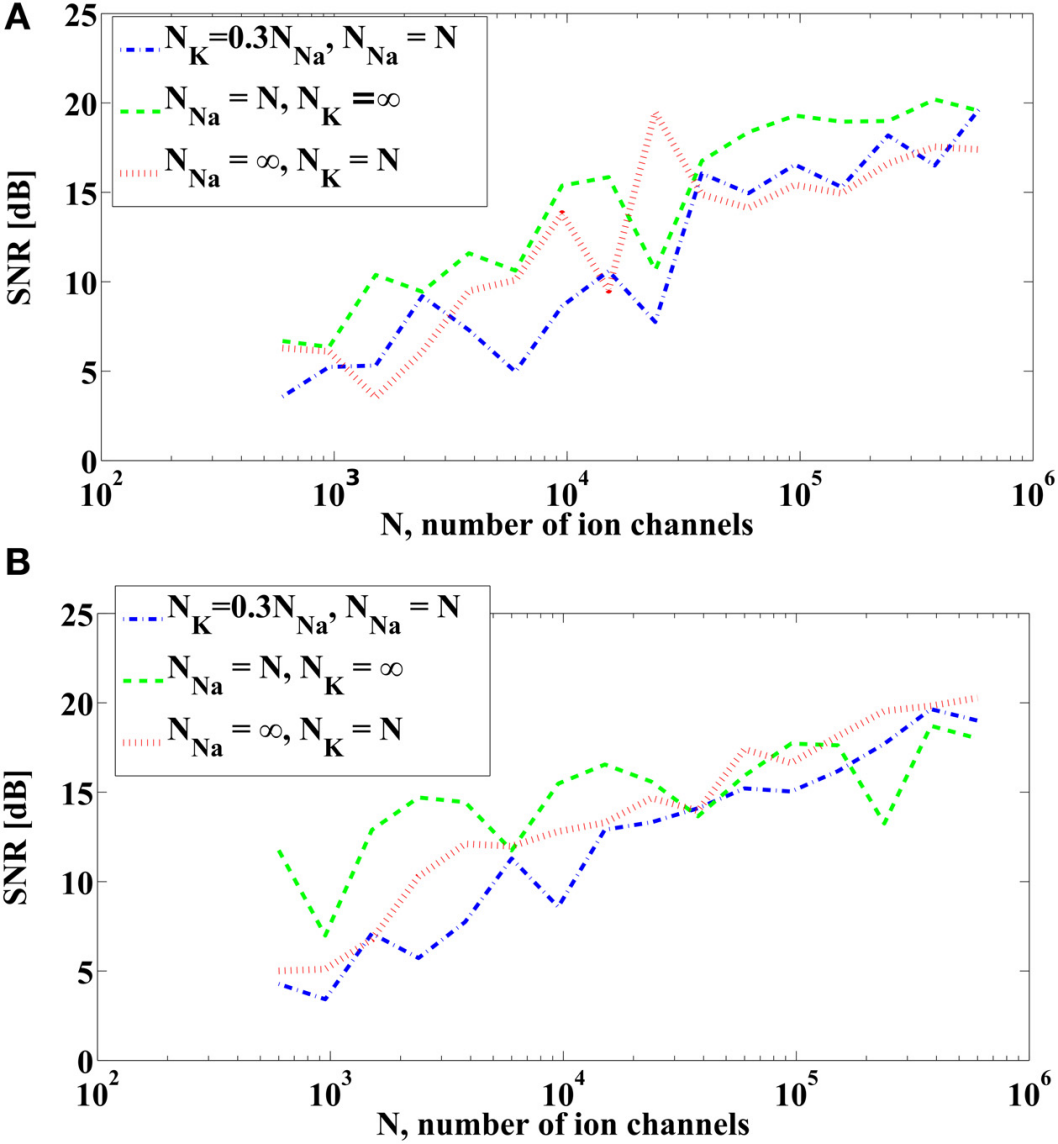
**SNR of reconstruction of the signals. (A)** SNR of *u*_1_(*t*). **(B)** SNR of *u*^2^_1_(*t*) = *u*_2_(*t*, *t*). (Blue) *N*_*Na*_ = *N*, *N*_*K*_ = 0.3*N*_*Na*_. (Green) *N*_*Na*_ = *N*, *N*_*K*_ = ∞. (Red) *N*_*Na*_ = ∞, *N*_*K*_ = *N*.

We now show how ion channel noise sources affect the decoding of the input signal. We varied the number of sodium channels *N*_*Na*_ and fixed the number of potassium channels to be *N*_*K*_ = 0.3*N*_*Na*_, a ratio typically used for Hodgkin-Huxley neurons with the alternative noise source model. By decoding the input stimulus we show how increasing the number of ion channels improves the faithfulness of signal representation. The SNR of the reconstruction of *u*_1_(*t*) and *u*^2^_1_(*t*) are depicted in Figure 6. SNR goes down to about 4 dB when 600 sodium channels and 180 potassium channels are used. This corresponds to a membrane area of about 10 μm^2^ with a density of 60 μm^2^ in sodium channels and 18 μm^2^ in potassium channels (Goldwyn et al., 2011). We also tested the reconstruction for the case when one type of ion channels is infinitely large, i.e., deterministic, while varying the number of ion channels of the other type. The result is also shown in Figure 6. The noise from the dendritic tree shall have similar effect on the representation since the voltage equation is the same as in Section 3.3.2.

## 4. Functional identification and noise

In Section 4.1 we show how to functionally identify the feedforward and feedback DSPs of the circuit in Figure 1 under noisy conditions. We assume here that the BSGs have already been identified using a methodology such as the one developed in Lazar and Slutskiy (2014). In Section 4.2 we discuss the effect of noise parameters on the quality of DSP identification.

### 4.1. Functional identification

In the decoding problem analyzed in Section 3.2, we reconstructed unknown input stimuli by assuming that the neural circuit in Figure 1 is known and the spike trains are observable. In contrast, the objective of the functional identification problem investigated in this section is to estimate the unknown circuit parameters of the feedforward and feedback DSPs from I/O data. The I/O data is obtained by stimulating the circuit with controllable inputs and by measuring the time occurrences of the output spikes. This basic methodology has been a standard practice in neurophysiology for inferring the function of sensory systems (Hubel and Wiesel, 1962). We assume here that either the BSGs are known in functional form or the family of PRCs associated with the BSGs have already been identified (Lazar and Slutskiy, 2014).

**Figure 7:**
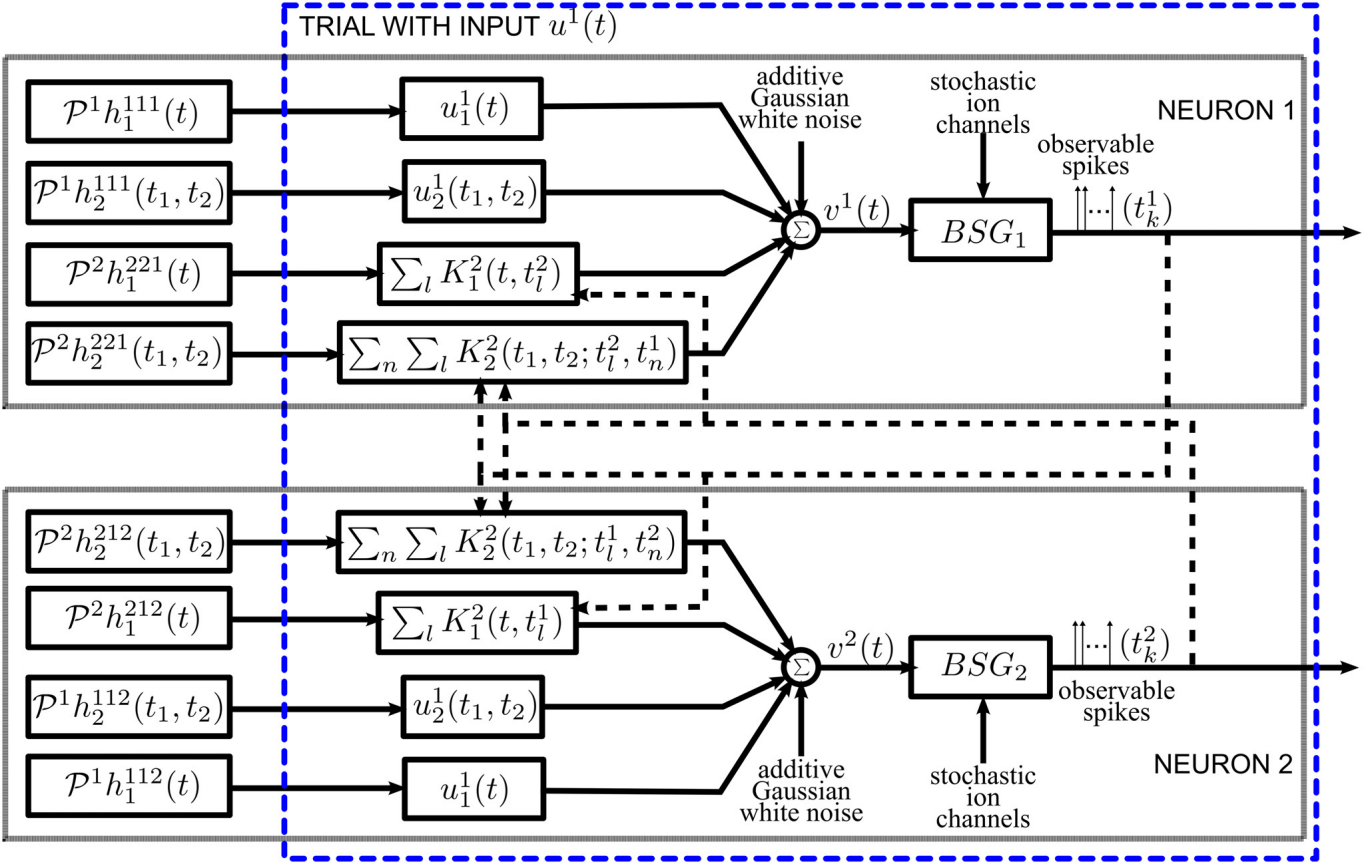
**Diagram of the neural circuit that is spike timing equivalent with the one in Figure 1 highlighting the duality between neural decoding and functional identification**. Note that the input stimuli and the DSP projections are reordered to reflect that the unknowns are the DSP projections. The input stimuli *u*^1^_1_(*t*), *u*^1^_2_(*t*_1_, *t*_2_), and the kernel representation of spikes (see also Section 2.2.2) are intrinsic to the neural circuit. The DSP projections are interpreted as inputs.

Although decoding and functional identification are seemingly two different problems, they are closely related. By exploiting the commutative property of linear operators, we can rearrange the diagram of the neural circuit model of Figure 1 into the form shown in Figure 7. We notice that the outputs of Figure 7 and those of Figure 1 are spike time equivalent, as long as the RKs *K*^2^_1_ and *K*^2^_2_ have large enough bandwidth. In what follows we will evaluate the four Volterra terms, i.e., the four dendritic currents feeding the BSG of Neuron 1 in Figure 7.

Formally, the first order (feedforward) Volterra term can be written as (Lazar and Slutskiy, in press)

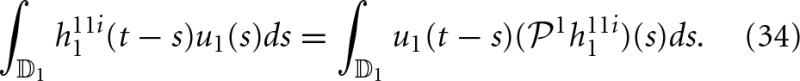



Similarly, the second order (feedforward) Volterra term amounts to

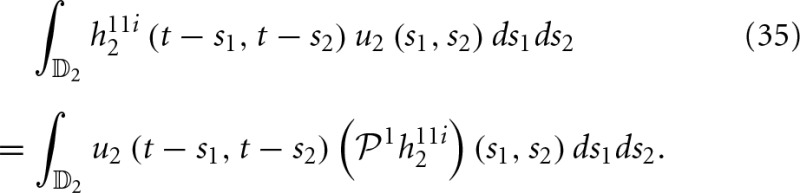



The above equations suggest that the projections of the feedforward kernels can be re-interpreted as inputs, whereas the signals *u*_1_ and *u*_2_ can be treated as feedforward kernels.

In Section 2.2.2 we introduced two RKHSs, 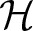
^2^_1_ and 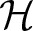
^2^_2_, for modeling two different spaces of spikes. The elements of 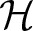
^2^_1_ are functions defined over the domain [0, *S*^2^] with

$$S^{2}\geq \mathrm{supp}\left\{h_{1}^{2ji}\right\}+\max{\left\{\left(t_{k+1}^{i}-t_{k}^{i}\right)\right\}}_{i=1,2,k\in \mathbb{Z}}.$$

The period *S*^2^ is large enough to ensure that any spike that arrives supp{*h*^2*ji*^_1_} seconds prior to the arrival of *t*^*i*^_*k*_, or earlier, will not affect the output of the feedback kernel on the inter-spike time interval [*t*^*i*^_*k*_, *t*^*i*^_*k* + 1_]. Thus, such spikes will not introduce additional error terms in the *t*-transform evaluated on the inter-spike time interval [*t*^*i*^_*k*_, *t*^*i*^_*k* + 1_]. Note that the domain [0, *S*^2^] of the functions in 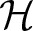
^2^_1_ may not be the same as the domain of the input space 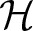
^1^_1_. However, such a domain can be shifted on a spike by spike basis to [*t*^*i*^_*k* + 1_ − *S*^2^, *t*^*i*^_*k* + 1_] for the inter-spike time interval [*t*^*i*^_*k*_, *t*^*i*^_*k* + 1_]. This is important for mitigating the practical limitation of modeling the stimuli as periodic functions in 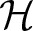
^1^_1_.

The response of the first-order feedback term to its spiking input on the inter-spike time interval [*t*^*i*^_*k*_, *t*^*i*^_*k* + 1_] in Figure 7 amounts to (*i* ≠ *j*)

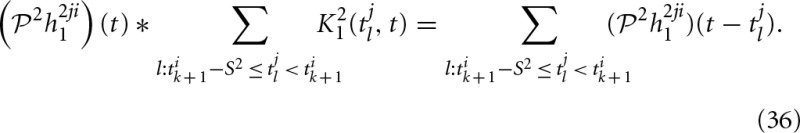



It is clear from Section 2.2.2 that

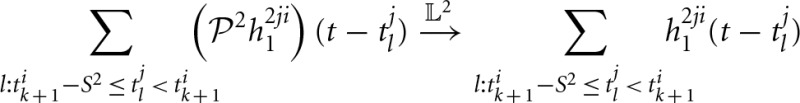



if Ω^2^ is at least larger than the effective bandwidth of *h*^2*ji*^_1_ and *L*^2^ → ∞.

Similarly, the response of the second-order feedback kernel to its spiking input on the inter-spike time interval [*t*^*i*^_*k*_, *t*^*i*^_*k* + 1_] amounts to

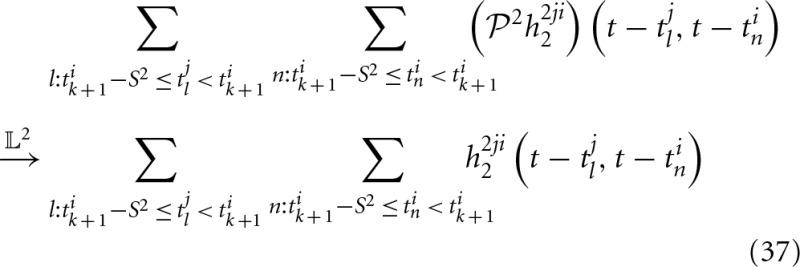



if Ω^2^ is large enough and *L*^2^ → ∞.

Combining (34), (36), (36), and (37), for each spike interval [*t*^*i*^_*k*_, *t*^*i*^_*k* + 1_], the aggregated output current of the DSPs of Neuron *i* in Figure 7, shall converge to the DSP aggregated output current of Neuron *i* in Figure 1 for large enough Ω^2^. A direct consequence of this equivalence is that, under the same additive Gaussian white noise and channel noise in the BSGs, the t-transform of the circuit in Figure 7 and in Figure 1 are identical.

Note that the outputs of the feedforward kernels are always equivalent; the equivalence of the outputs of the feedback kernels requires, however, the use of large enough bandwidth Ω^2^. Otherwise, the equivalence in the t-transform is invalid and an additional noise term appears in the *t*-transform of the Neuron 1 in Figure 7.

**Figure 8:**
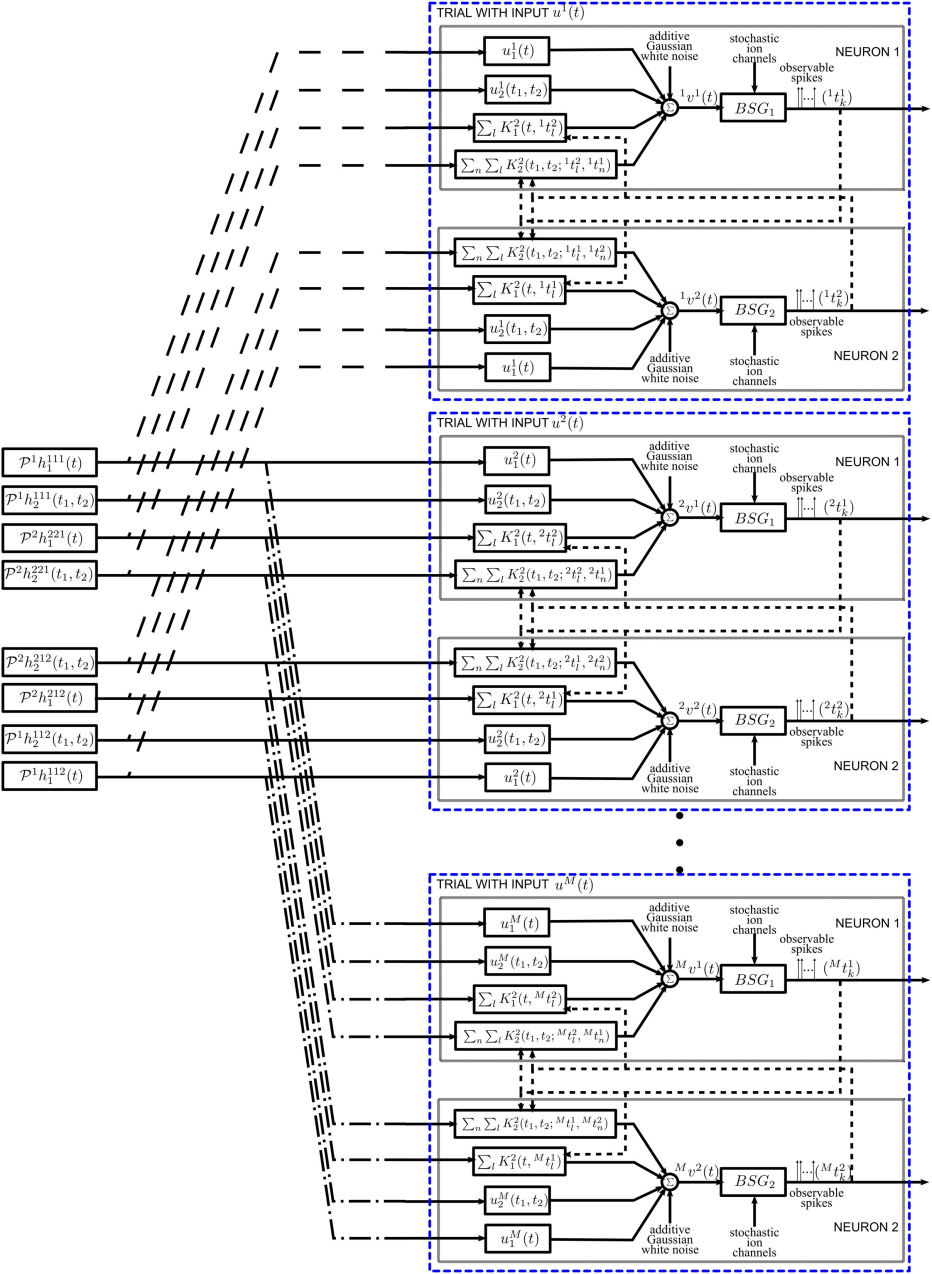
**Diagram of the functional identification with multiple trials**. The neural circuit is presented a different stimulus *u*^*m*^_1_(*t*) for each trial *m*. See also Figure 7 for details of a single trial.

The projections of the Volterra DSP kernels of Figure 7 are interpreted as inputs, while the input stimuli and the train of RKs at spike times replace the impulse response of the corresponding filters. Therefore, the functional identification problem has been transformed into a dual decoding problem, where the objects to decode are the set of projections of Volterra DSP kernels and the neural circuit is comprised of “stimulus DSP kernels” and “spike DSP kernels” with the same BSGs and noise sources. The only difference is that, instead of a Single-Input Multi-Output decoding problem, the identification was transformed into a Multi-Input Multi-Output decoding problem. In addition, multiple trials using different stimuli are needed; this procedure is illustrated in block diagram form in Figure 8. By stimulating the neural circuit with multiple stimuli in the functional identification setting, multiple neural circuits effectively encode the projections of the DSP kernels.

We are now in the position to derive the t-transform of Neuron 1 in Figure 7. Assuming that *m* = 1, …, *M*, trials are performed for identification, the *t*-transform (26) can be written as

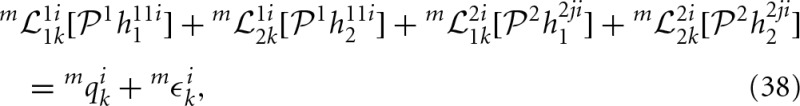



for *i*, *j* = 1, 2, *i* ≠ *j*, *k* ∈ ℤ. Here ^*m*^
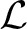
^1*i*^_1*k*_: 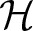
^1^_1_ → ℝ, ^*m*^
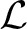
^1*i*^_2*k*_: 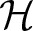
^1^_2_ → ℝ are bounded linear functionals associated with the feedforward DSP kernels, and ^*m*^
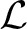
^2*i*^_1*k*_: 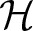
^2^_1_ → ℝ, ^*m*^
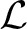
^2*i*^_2*k*_: 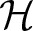
^2^_2_ → ℝ are bounded linear functionals associated with the feedback DSP kernels for each trial *m*. The above functionals are defined as

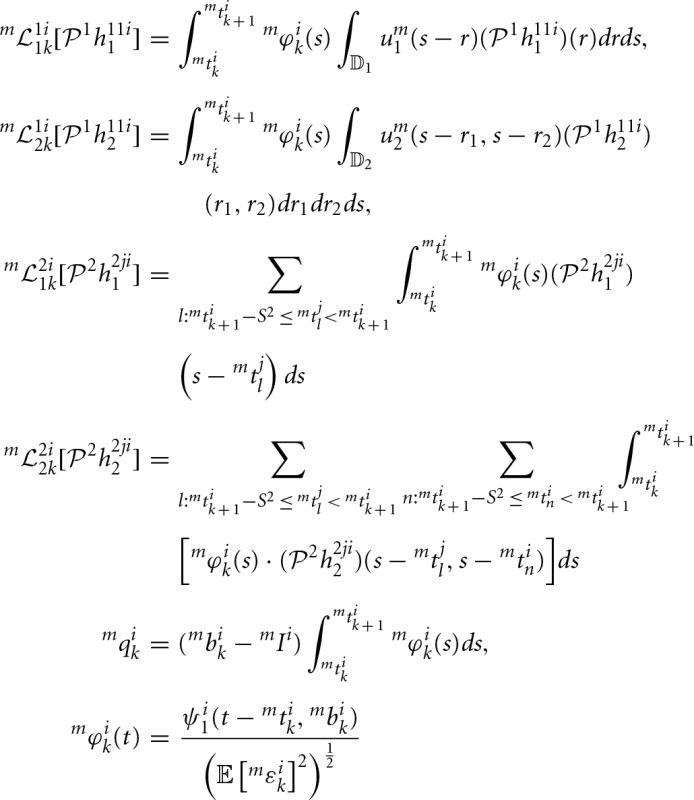



and ^*m*^ϵ^*i*^_*k*_, *i* = 1, 2, *k* ∈ ℤ, *m* = 1, …, *M*, are independent random variables with normal distribution 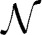
(0, 1).

The functional identification of the neural circuit in Figure 7 can then be similarly defined to the decoding problem. We formulate the identification of the noisy neural circuit again as two smoothing spline problems, one for each neuron,

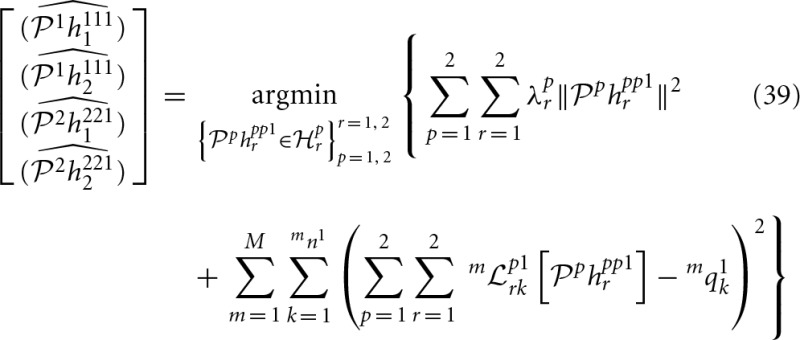



and

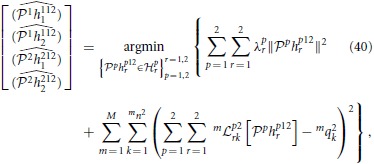



where ^*m*^*n*_*i*_ is the number of spikes generated by Neuron *i* in trial *m*.

The solution can be obtained in a similar way as in Theorem 3.7.

**Theorem 4.1**. *The solutions to* (40) *is*

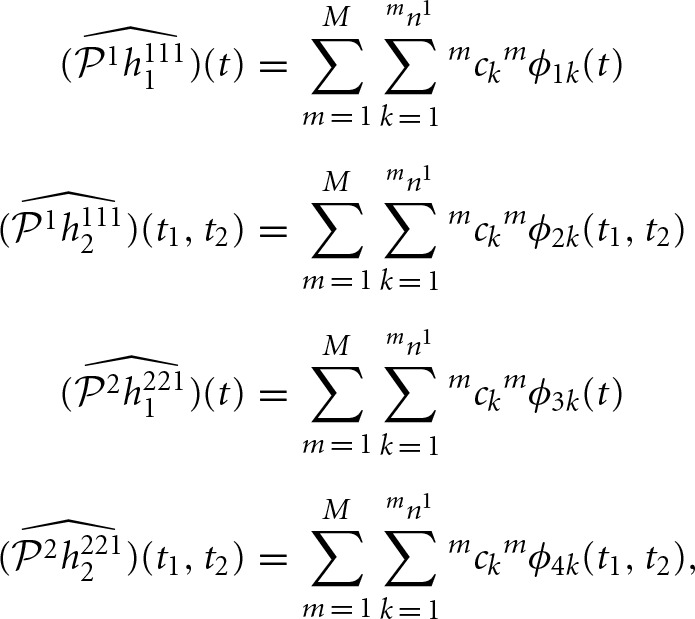



where

$$c={\left[{}^{1}c_{1}\cdots^{1}c_{{}^{1}n^{1}},\cdots ,\cdots {,}^{M}c_{1}\cdots^{M}c_{{}^{M}n^{1}}\right]}^{T},$$

is the solution to the system of linear equations

(41) $$\begin{array}{l} \left({\left(\Phi_{1}+\Phi_{2}+\Phi_{3}+\Phi_{4}\right)}^{2}+\lambda_{1}^{1}\Phi_{1}+\lambda_{1}^{2}\Phi_{2}+\lambda_{2}^{1}\Phi_{3}+\lambda_{2}^{2}\Phi_{4}\right)c\\ =\left(\Phi_{1}+\Phi_{2}+\Phi_{3}+\Phi_{4}\right)q, \end{array}$$

where

$$q={\left[{}^{1}q_{1}^{1}\cdots^{1}q_{{}^{1}n^{1}}^{1},\cdots ,\cdots {,}^{M}q_{1}^{1}\cdots^{M}q_{{}^{M}n^{1}}^{1}\right]}^{T},$$

and

$$\Phi_{i}=\left[\begin{array}{c} \begin{array}{l} \Phi_{i}^{11}\;\cdots \;\Phi_{i}^{1M}\\ \;\vdots \;\ddots \;\vdots \\ \Phi_{i}^{M1}\cdots \Phi_{i}^{MM}, \end{array} \end{array}\right]$$

and finally

$${\left[\Phi_{i}^{mn}\right]}_{kl}=\langle^{m}\phi_{ik}{,}^{n}\phi_{il}\rangle .$$

*In addition, the sampling functions* ^*m*^ϕ_*ik*_ *are given by*

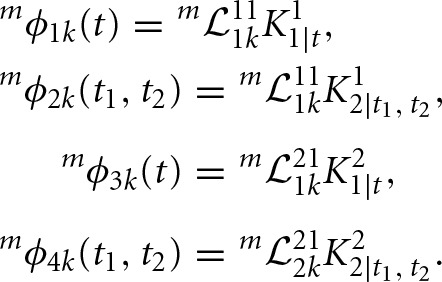



**Proof:** The proof is similar to the one of Theorem 3.7.    □

Since each of the kernel projections may be in a different RKHS, and their domain may also be different, the identification of all filters resemble that of the multi-sensory Time Encoding Machines. Recall that multi-sensory TEMs encode within the same circuit time-varying and space-time varying sensory signals while decoding remains tractable (Lazar and Slutskiy, 2013). The solution to (41) can similarly be obtained as the solution to (40) above.

Note that we are only able to identify the projection of the Volterra kernels. This is because, in practice, we can only probe the system with signals in a bandlimited space. By increasing the bandwidth of the elements of the Hilbert space, the projection of the kernels will converge to their original form (Lazar and Slutskiy, 2012).

**Remark 4.2**. *It is important to note that in order to have a good estimate of the kernels, stimuli must fully explore all input spaces. This can be quite easily achieved for the feedforward DSP kernels by using many (randomly generated) signals that cover the entire frequency spectrum. However, to properly identify the feedback DSP kernels, spike trains must be diverse enough to sample its different frequency components. This may not be easy to realize in practice. For first order feedback kernels, spike trains with constant spike intervals are, for example, undesirable. The Fourier transform of regular Dirac-delta pulses is a train of Dirac-delta pulses in the Fourier domain. This means that only certain frequency responses of the DSP kernels are, for example the DC component, sampled. The rest of the frequency components are essentially in the null space of the sampling functions* ^*m*^ϕ_*ik*_, *i* = 1, 2, *m* = 1, …, *M*. *Similar effect applies to the space of trigonometric polynomials. If the spike intervals exhibit small variations, many of the frequency components may be sampled but the energy at DC may be too dominant. In this case, noise may contaminate more severely the measurement of non-DC components and may yield unsatisfactory identification. This effect may pose even more stringent requirements on the identification of the second order feedback kernels, as it requires the interaction between two spike trains*.

### 4.2. Effect of noise on identification

In order to evaluate the effect of noise on the identification of the neural circuit in Figure 1 we included intrinsic noise into the example neural circuit discussed under noiseless conditions in Section 4.1 of the Supplementary Material. Randomly generated signals were used in the identification examples given here. Chosen in the same way as in the decoding example in Section 3.3.2 all these signals are used here to identify the neuron in question. Therefore, in this section, multiple signals are used in repeat experiments to identify the parameters of a neural circuit. By contrast in Section 3.3.2, multiple neurons are used to encode a single signal.

**Figure 9:**
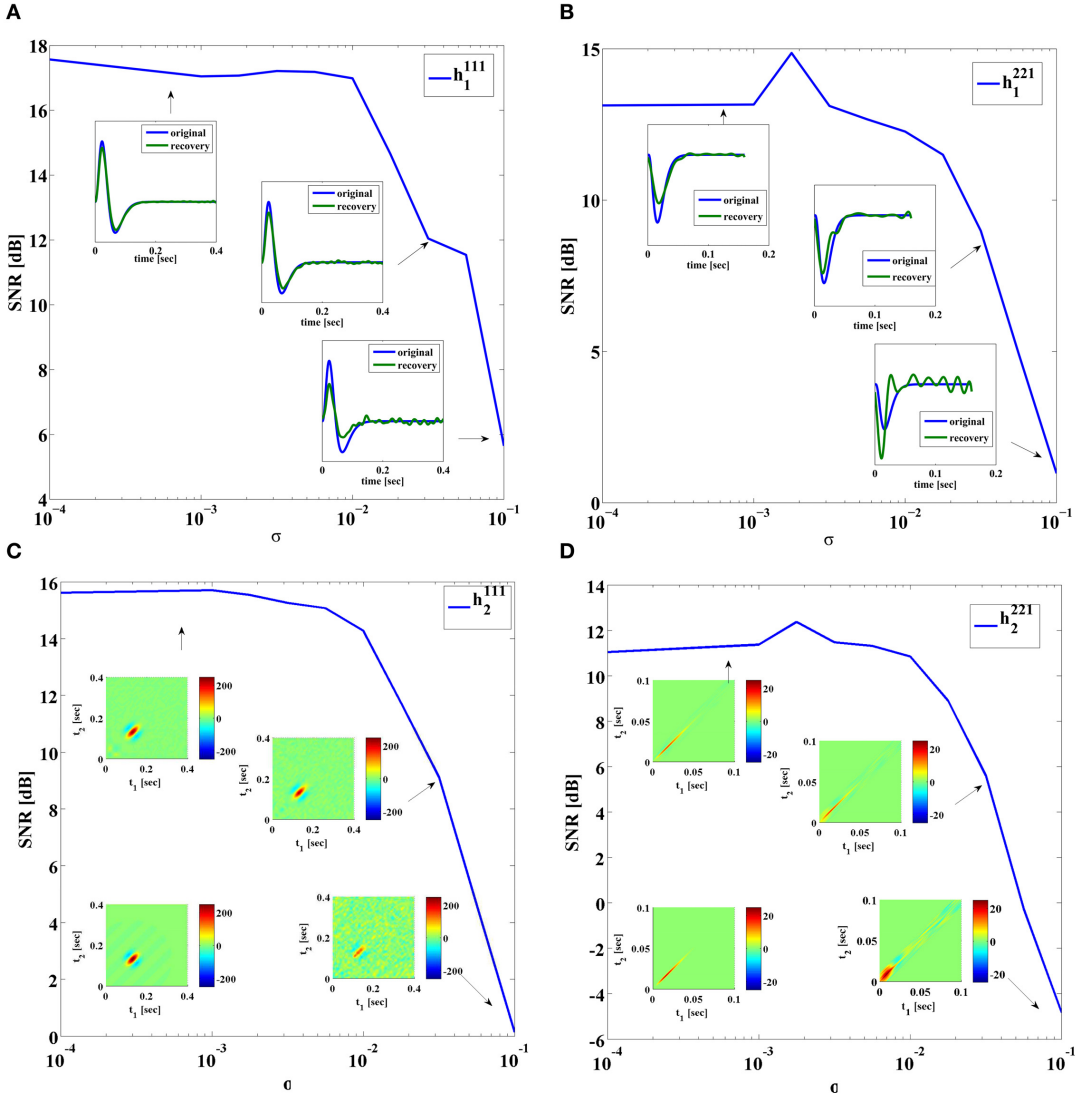
**SNR of identified DSP kernels**. Noise added using SDE (31), with 10σ^*i*^_1_ = 10σ^*i*^_2_ = σ^*i*^_3_ = σ^*i*^_4_ = σ. **(A)** Kernel *h*^111^_1_. In-sets provide a comparison between the original and the identified kernel. **(B)** Kernel *h*^111^_2_. In-sets are identified kernels. Original kernel is on the lower left. **(C)** Kernel *h*^221^_1_. In-sets provide a comparison between the original and the identified kernel. **(D)** Kernel *h*^221^_2_. In-sets are identified kernels. Original kernel is on the lower left.

First, we evaluated the effect of noise on the quality of identification of DSP kernels of Neuron 1 in Figure 7 with a BSG modeled by the SDE (31) with 10σ^*i*^_1_ = 10σ^*i*^_2_ = σ^*i*^_3_ = σ^*i*^_4_ = σ. Figure 9 shows the SNR of the identified DSP kernels in Figure 7 across several noise levels σ. As expected, the general trend for all four kernels is decreasing SNR with increasing noise levels. We notice that the identified feedforward DSP kernels have similar shape as the original kernel, even at high noise levels. However, the feedback DSP kernels undergo a change in shape at high noise levels. We can see that the time instance of the peak amplitude in the first order feedback kernel is shifted to an earlier time instance.

**Figure 10:**
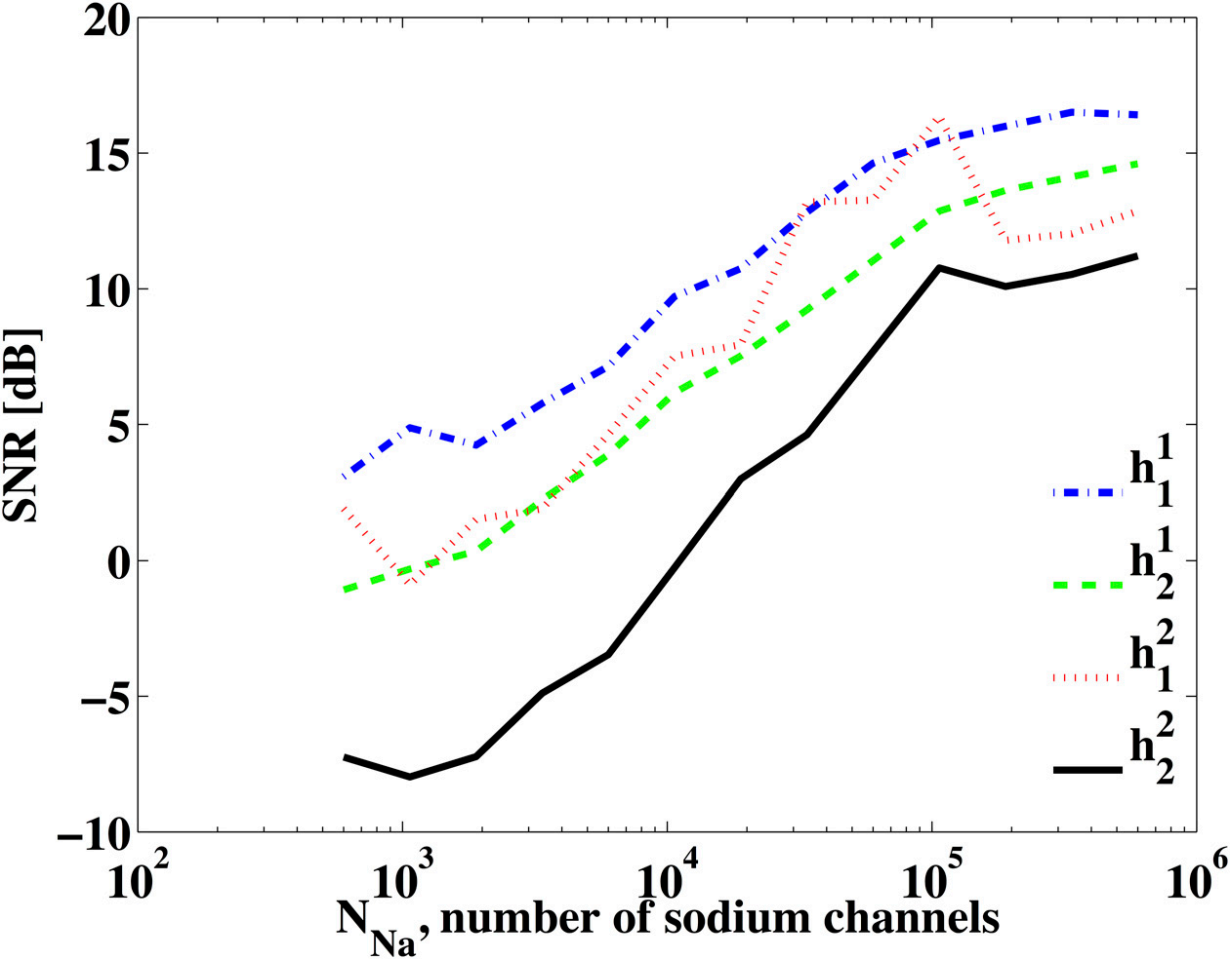
**SNR of identified DSP kernels**. The BSG is described by the Hodgkin-Huxley equations with a finite number of ion channels and *N*_*K*_ = 0.3*N*_*Na*_.

Second, we investigated the identification of DSPs for a BSG noise model already described in Section 3.3.3. Figure 10 shows the SNR of the identified DSP kernels across a different number of sodium channels *N*_*Na*_ while *N*_*K*_ = 0.3*N*_*Na*_. The SNR plots suggest that the identification quality increases as more ion channels are present in the BSGs.

**Figure 11:**
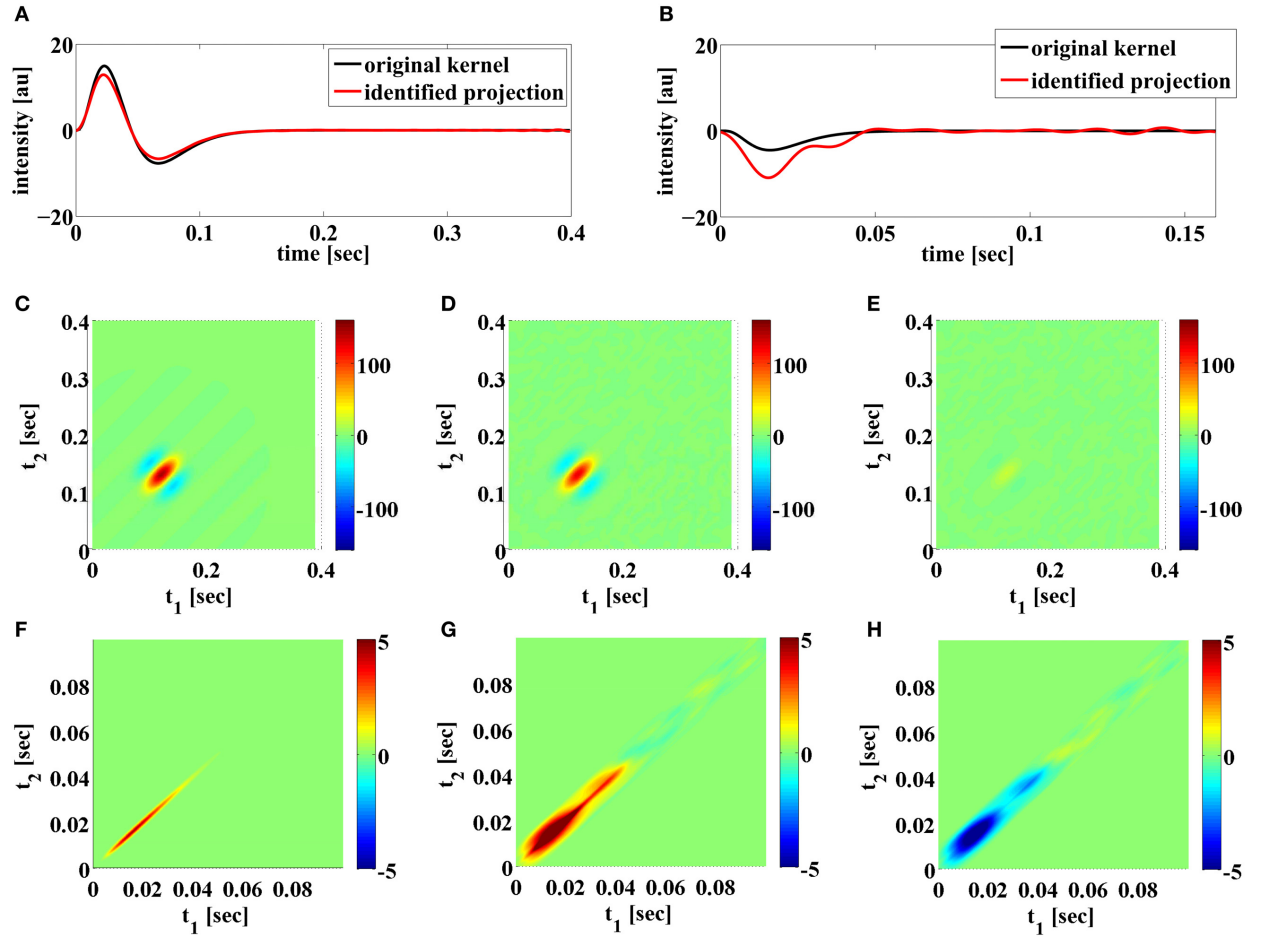
**Examples of functional identification when the generated spikes do not fully explore the space of feedback kernels. (A)** Original first order feedforward kernel (black) and identified projection of the kernel (red). **(B)** Original first order feedback kernel (black) and identified projection of the kernel (red). **(C)** Original second order feedforward kernel. **(D)** Identified projection of second order feedforward kernel. **(E)** Error of identified second order feedforward kernel. **(F)** Original second order feedback kernel. **(G)** Identified projection of second order feedback kernel. **(H)** Error of identified second order feedback kernel.

Additionally, as discussed in Remark 4.2, BSG noise sources may degrade severely the identification of feedback kernels when the spike trains generated in trials are not sufficient for exploring the two spike input spaces. We show an example of the later in Figure 11. The two BSGs have higher bias currents and lower input current magnitude. The later was achieved by scaling down the magnitude of the DSP kernels. The combined effect results in regular spiking intervals in both neurons. The identification result under *noiseless conditions* is shown in Figure 11. Note that since the *t*-transform of the Hodgkin-Huxley neuron is not exact, a small error is introduced even if intrinsic noise is not present. We see that the feedforward DSP kernels can be identified quite well, yielding SNRs of around 17 dB. However, the feedback DSP kernels are not well identified. In particular, the identified second-order feedback kernel has a wide spread, similar to the high noise case in Figure 9D. This suggest that the spike pattern is not sufficiently exploring the space of feedback kernels. A large number of frequency components are only weakly sampled and they can be very easily contaminated by noise. The presence of both intrinsic noise sources can exacerbate the condition further. This effect is confirmed with a simulation of the circuit using Integrate-and-Fire (IAF) neurons. Since the *t*-transform for the IAF neuron is exact (Lazar and Tóth, 2004), both feedback kernels can be identified even if the generated spikes only weakly explore certain frequency components. However, by artificially adding a small measurement error to the t-transform of the circuit with IAF neurons, similar results to those in Figure 11 can be obtained (data not shown).

## 5. Discussion

In this paper, we introduced a novel neural circuit architecture based on a neuron model with a biophysical mechanism of spike generation and feedforward as well as feedback dendritic stimulus processors with intrinsic noise sources. Under this architectural framework, we quantitatively studied the effect of intrinsic noise on dendritic stimulus processing and on spike generation. We investigated how intrinsic noise sources affect the stimulus representation by decoding encoded stimuli from spikes, and quantified the effect of noise on the functional identification of neural circuits. We argued that a duality between stimulus decoding and functional identification holds. Therefore, the encoding framework based on the neural circuit architecture studied here can be applied to both the reconstruction of the encoded signal and the identification of the dendritic stimulus processors. We systematically showed how the precision in decoding is affected by different levels of stochastic variability within the circuit. These results apply verbatim to the functional identification of dendritic stimulus processors via the key duality property mentioned above.

Our theoretical framework highlights two indispensable components of modeling signal representation/processing in a neural circuit—dendritic stimulus processing and spike generation. Such a divide and conquer strategy is ubiquitous in engineering circuits and leads to a separation of concerns. Recent experimental studies also showed that interesting nonlinear processing effects take place first in the dendritic trees rather than in the axon hillock (Yonehara et al., 2013).

We presented here two types of nonlinear dendritic stimulus processors. The first type are feedforward DSPs that respond to continuous input sensory stimuli. The second type are feedback DSPs that respond to *spiking* inputs. We quantitatively demonstrated how intrinsic noise sources would affect the identification quality of all these DSPs. The examples in Section 4.2 suggest that in identification feedback kernels are more vulnerable to internal noise sources than feedforward kernels. In particular, the overall shape of the identified feedback kernels differs significantly from that of the underlying kernels when the strength of noise sources becomes large. Meanwhile the identified feedforward kernels are qualitatively preserved, albeit not accurately.

Most of the single neuron models (LIF, QIF) in the literature have focused on the spike generation mechanism. The encoding capability of these models is typically investigated based on rate encoding (Eliasmith and Anderson, 2003; Lundstrom et al., 2008; Ostojic and Brunel, 2011). For both decoding and identification we used here the occurrence times of spikes generated by spiking neuron models. Most importantly, the BSG models discussed here were characterized by a PRC manifold (Kim and Lazar, 2012) in the presence of noise, while many simplified models (such as the LIF) can be effectively described with a single PRC. Other sensory neuron models, e.g., GLM (Pillow et al., 2011), usually rely on a rate-based output or Poisson spike generation that do not take into account key advances in dynamical systems-based spiking neuron models.

As already mentioned before, we investigated how intrinsic noise sources affect the stimulus representation by decoding encoded stimuli from spikes. We are not suggesting, however, that the decoding algorithm considered here is implemented in the brain. Rather, we argue that decoding is effective in measuring how well information is preserved in the spike domain. The decoding formalism allowed us to investigate how noise affects the fidelity of signal representation by a population of neurons by reconstructing stimuli and comparing their information content in the stimulus space.

While decoding can serve as an “oscilloscope” in understanding stimulus representation in sensory systems, functional identification serves as a guide in experiments to functionally identify sensory processing. Based on spike times, the identification algorithm presents a clear bound on the number of spikes that are necessary for perfect identification under noiseless conditions. Phrased differently, when a certain number of spikes are acquired from a neuron of interest, the identification algorithm places a constraint on the maximum DSP kernel bandwidth that can perfectly be recovered.

In more practical terms, we advanced two important applications of the circuit architecture considered in this paper. The first one considers dendritic stimulus processors that process information akin to complex cells in V1. The second one adapts the widely used Hodgkin-Huxley model known in the context of neural excitability (Izhikevich, 2007) and analysis of neuronal stochastic variability to stimulus encoding in the presence of noise.

Based on the rigorous formalism of TEMs (Lazar and Tóth, 2004), we extended our previous theoretical framework (Lazar et al., 2010) and argued that spike timing is merely a form of generalized sampling of stimuli. By studying sampling (or measurements) in the presence of intrinsic noise sources, we showed to what extent neurons can represent sensory stimuli in noisy environments as well as how much noise the identification process can tolerate while preserving an accurate understanding of circuit dynamics.

The reconstruction and identification quality are certainly not only related to the strength of noise, but also the strength of the signal. In particular, when the signal strength is small, two facts may affect the quality of reconstruction. First, neurons may not produce enough spikes that have valid *t*-transforms. Second, they may be contaminated by even weak noise, i.e., the signal-to-noise ratio is low. It is well known, however, that neural systems use gain control to boost the relevant signal (Shapley and Victor, 1978; Wark et al., 2007; Friederich et al., 2013). Such strategy may be useful for increasing the signal strength relatively to the strength of the noise. Gain control may also suppress large signals to fit into the range of operation of the BSGs. The gain control itself, maybe considered as a type of Volterra feedforward DSP kernel (Lazar and Slutskiy, in press) and the interaction with feedback loops driven by spikes. The lack of spikes may be compensated by adding other neurons that are sensitive to other features in the input stimuli.

A key feature in our neural circuit model is the nonlinear processing in the feedforward and feedback paths. Nonlinear interaction between feedforward DSPs and feedback DSPs have not been considered here. However, they are of interest and could be addressed in the future. Self-feedback was not included in the model for clarity, but can be considered within the framework of our approach. Self-feedback was introduced to add refractoriness to phenomenological neuron models (Keat et al., 2001; Pillow et al., 2008). Our BSG models, on the contrary, are conductance-based models that have a refractory period built in.

Throughout this paper we assumed that the BSGs themselves have been perfectly identified. The intrinsic noise in the BSGs may degrade the identification quality of conditional PRCs. This may result in a lower identification quality as shown in the examples. It is beneficial to investigate in the future a method that can identify the entire circuit at once so that intrinsic noise in the circuit only affects the identification process a single time.

The theoretical results obtained here suggest a number of experiments in the early olfactory system of fruit flies. The glomeruli of the antennal lobe can be modeled using the Volterra DSPs discussed here and the projection neurons in the antennal lobe are accessible by patch clamping (Lazar and Yeh, 2014). Functional identification of DSPs can then be carried out for studying olfactory stimulus processing in an accessible circuit with intrinsic noise sources (Masse et al., 2009).

## Conflict of interest statement

The authors declare that the research was conducted in the absence of any commercial or financial relationships that could be construed as a potential conflict of interest.

## Appendix

### Proof of theorem 3.7

**Proof:** By the Riesz representation theorem (Berlinet and Thomas-Agnan, 2004), there exists a function ϕ^*i*^_1*k*_ ∈ ^1^_1_ such that ^*i*^_1*k*_*u*_1_ = 〈*u*_1_, ϕ^*i*^_1*k*_〉, ∀*u*_1_ ∈ ^1^_1_. Moreover by the reproducing property

Let ^1^_10_ be a linear subspace of ^1^_1_ spanned by ϕ^*i*^_1*k*_

and let ^1⊥^_10_ be a linear subspace of ^1^_1_ defined by

Then, for any *u*_1_ ∈ ^1⊥^_10_ and any $\sum_{i\;=\;1}^{2}\sum_{k\;=\;1}^{n^{i}}c_{k}^{i}\phi_{1k}^{i}$ ∈ ^1^_10_, we have

Since ^1^_1_ = ^1^_10_ ⊕ ^1⊥^_10_, *u*_1_ can be represented as *u*_1_ = *u*_10_ + *u*^⊥^_10_ where *u*_10_ ∈ ^1^_10_ and *u*^⊥^_10_ ∈ ^1⊥^_10_ are orthogonal. Therefore,

$$\|u_{10}+u_{10}^{⊥}{\|}^{2}=\|u_{10}{\|}^{2}+\|u_{10}^{⊥}{\|}^{2}.$$

Similarly, there exists a function ϕ^*i*^_2*k*_ ∈ ^1^_2_ such that ^*i*^_2*k*_*u*_2_ = 〈*u*_2_, ϕ^*i*^_2*k*_〉, where ϕ^*i*^_2*k*_(*t*_1_, *t*_2_) = ^*i*^_2*k*_*K*^1^_2|*t*_1_*t*_2__. *u*_2_ can be represented as *u*_2_ = *u*_20_ + *u*^⊥^_20_, where *u*_20_ ∈ ^1^_20_ and *u*^⊥^_20_ ∈ ^1⊥^_20_ are orthogonal, with

and

Finally,

Therefore, the minimizer to (28) must belong to the subspaces ^1^_10_ and ^1^_20_.

By plugging (29) into (28) and setting the gradient with respect to **c** to 0, we see that **c** is the solution to (30).    □
