# Supplementary material for: Volterra dendritic stimulus processors and biophysical spike generators with intrinsic noise sources
Source: Front Comput Neurosci. 2014 Sep 1;8:95. doi: 10.3389/fncom.2014.00095 (PMC4150400; doi:10.3389/fncom.2014.00095)
Supplement: Supplementary file 1 [file DataSheet1.PDF]

Supplementary Material: Volterra Dendritic Stimulus Processors and Biophysical Spike Generators with Intrinsic Noise Sources

Aurel A Lazar [1,*], Yiyin Zhou [1]

[1] *Department of Electrical Engineering, Columbia University, New York, NY, USA*

Correspondence*:
Aurel A. Lazar
Department of Electrical Engineering, Columbia University, 500 W. 120th Street, New York, NY 10027, USA, aurel@ee.columbia.edu

Author's names are alphabetically listed.

Neuronal stochastic variability: influences on spiking dynamics and network activity

1 ORGANIZATION OF THE SUPPLEMENTARY MATERIAL

The supplementary material presented here is organized according to the section in which they are referenced in the main article.

2 SUPPLEMENTARY MATERIAL FOR SECTION 2

2.1 DIAGRAM OF PARALLEL ENCODING CIRCUIT

Supplementary Figure 1 shows a Single-Input Multiple-Output (SIMO) encoding diagram that consists of M of the neural circuits in Figure 1. They simultaneously encode common stimulus $u_1(t)$.

2.2 EXAMPLE OF BSG: HODGKIN-HUXLEY NEURON

We consider a Hodgkin-Huxley neuron with standard parameters, described by the non-linear differential equations:

$$\begin{aligned} C\frac{dV}{dt} &= -\bar{g}_{Na}m^3h(V-E_{Na}) - \bar{g}_K n^4(V-E_K) - \bar{g}_L(V-E_L) + I \\ \frac{dn}{dt} &= \alpha_n(V)(1-n) - \beta_n(V)n \\ \frac{dm}{dt} &= \alpha_m(V)(1-m) - \beta_m(V)m \\ \frac{dh}{dt} &= \alpha_h(V)(1-h) - \beta_h(V)h \end{aligned} \quad , \tag{S1}$$

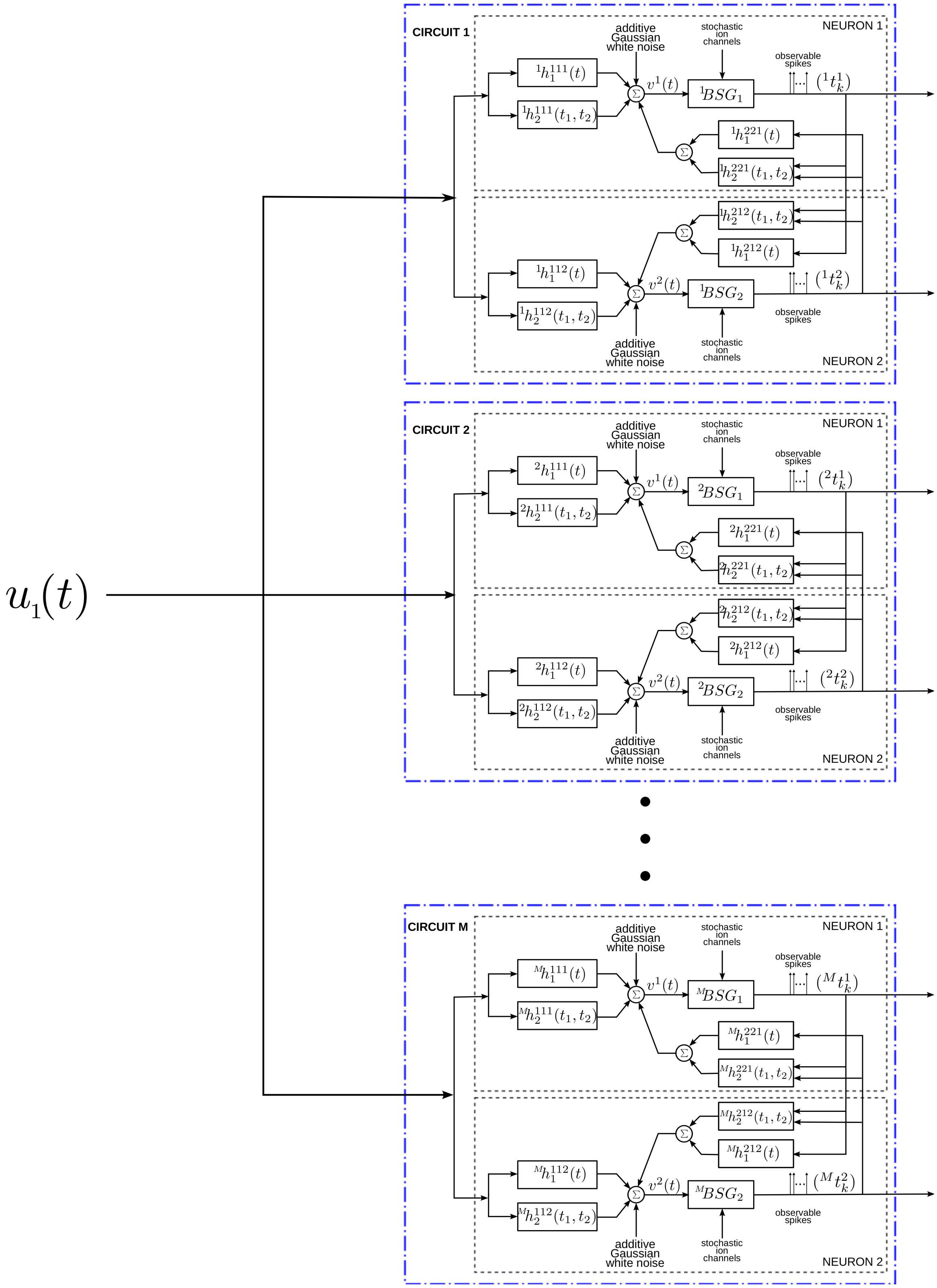

Supplementary Figure 1: Single-Input Multi-Output (SIMO) encoding diagram with, in parallel, M of the neural circuits in Figure 1 that simultaneously encode input $u_1(t)$.

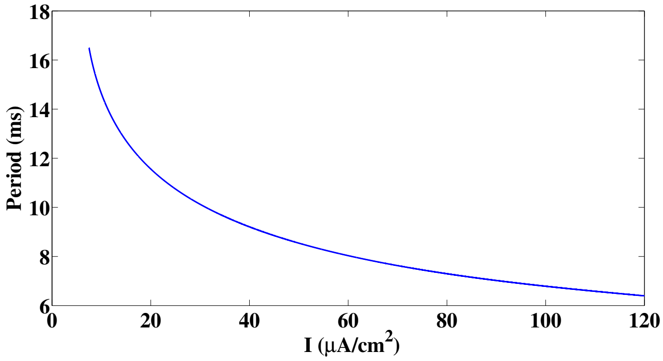

Supplementary Figure 2: Period of oscillation when Hodgkin-Huxley neuron is subject to a constant current I.

and

$$\begin{aligned} \alpha_n(V) &= \frac{0.01(V+55)}{1-e^{-\frac{V+55}{10}}} \\ \beta_n(V) &= 0.125e^{-\frac{V+65}{80}} \\ \alpha_m(V) &= \frac{0.1(V+40)}{1-e^{-\frac{V+40}{10}}} \\ \beta_m(V) &= 4e^{-\frac{V+65}{18}} \\ \alpha_h(V) &= 0.07e^{-\frac{V+65}{20}} \\ \beta_h(V) &= \frac{1}{1+e^{-\frac{V+35}{18}}} \end{aligned}, \tag{S2}$$

where V is the membrane potential, n, m, h are the gating variables, and I is the bias current. The latter is assumed to be large enough to induce periodic spiking. Therefore, the Hodgkin-Huxley neuron considered here is a periodically spiking neuron with period $T(I)$, where $T = T(I)$ maps the bias current I into the period of spiking T. The function T is shown in Supplementary Figure 2. In other words, $T(I)$ is closely associated with the $f - I$ curve typically seen in the literature. Without loss of generality, we will assume for simplicity that $C = 1\mu F/cm^2$. Since the Hodgkin-Huxley neuron is periodically spiking, it has a well-defined PRC $\boldsymbol{\psi}(t, I) = [\psi_1(t, I), \psi_2(t, I), \psi_3(t, I), \psi_4(t, I)]^T$, where $\psi_1(t, I), \psi_2(t, I), \psi_3(t, I), \psi_4(t, I)$ are the PRCs associated with the component states V, n, m, h, respectively. There are multiple ways of evaluating the PRC of a periodically spiking neuron with weak coupling, among which Malkin's method is numerically efficient (see **Lazar** (2010); **Izhikevich** (2007)).

2.3 EXAMPLE OF SPIKES GENERATED BY THE NEURAL CIRCUIT WITH NOISE SOURCES

An example of raster plot of the output spikes generated by the two neurons subject to 50 trials of the same stimulus is shown in Supplementary Figure 3. We used the feedforward kernels of Example 2.5 and set the feedback kernels to be zero. Hodgkin-Huxley neurons in Supplementary Section 2.2 are used for

BSGs with noise added as in (16). We set $\mathbf{B^i} = \mathbf{I}$ and

$$d\mathbf{Z}^i = \begin{bmatrix} v^i dt + \sigma dW_1^i \\ \sigma dW_2^i \\ \sigma dW_3^i \\ \sigma dW_4^i \end{bmatrix},$$

where the scaling factor $\sigma = 0.01$. Since the initial conditions were the same for each trial, we see that initially the spikes are closer to each other across trials. As time progresses, the variability in spike times is increasing and is clearly visible.

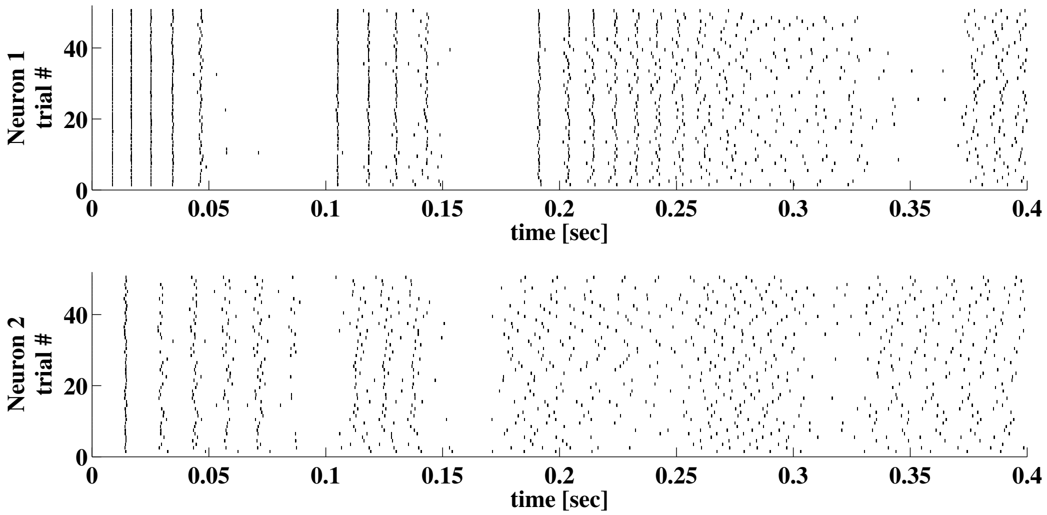

Supplementary Figure 3: Raster plot of spikes generated by the neural circuit of Figure 1. The same stimulus is applied 50 times and the spike times are recorded for both neurons. The feedforward kernels employed in Example 2.5 were used and the feedback kernels were set to zero. The Hodgkin-Huxley neurons described in Supplementary Section 2.2 are used for the BSGs. Variability in spike timing is clearly visible with repeated presentation of the same stimulus. Note that the repeated trials here are only for the purpose of demonstrating variability in spike timing due to intrinsic noise sources in the neural circuit. In the formulation of the stimulus encoding/decoding problem in Section 3, the decoding algorithm only requires each stimulus to be presented to the neural circuit a single time.

3 SUPPLEMENTARY MATERIAL FOR SECTION 3

3.1 CHOOSING SPIKE INTERVALS

We provide a simple example of encoding by a Hodgkin-Huxley neuron. The input to the Hodgkin-Huxley neuron is shown in the Supplementary Figure 4 (blue curve), while the spikes generated by the neuron in response to the input are indicated by stems. The spike intervals between red stems are deemed valid. All other spike intervals are discarded in decoding and identification. We see that most of the discarded spike intervals correspond to a low input current.

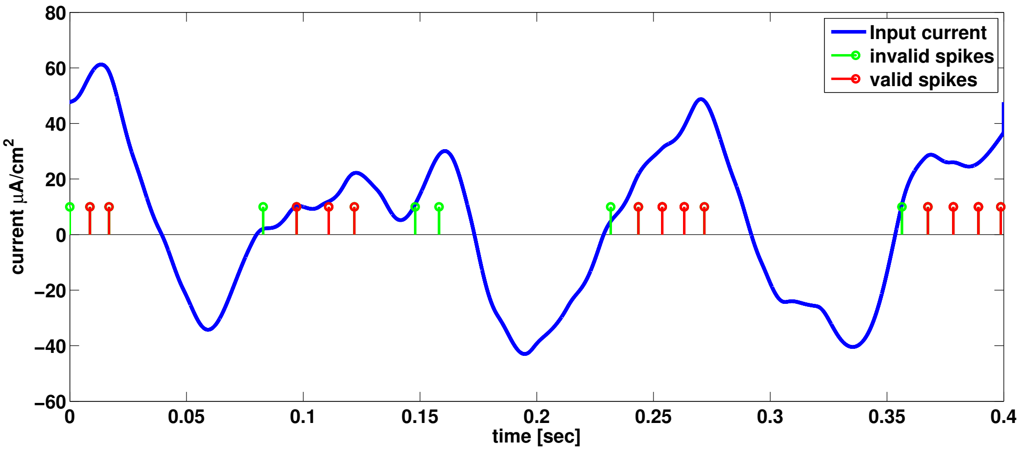

Supplementary Figure 4: Example of input to a BSG and spikes generated. An Hodgkin-Huxley neuron, described in Supplementary Section 2.2, was injected with a time-varying current (blue curve). 19 spikes were generated in response to the injected current. We only consider the t-transform on time intervals between spikes that are both labeled with red stems. The other inter-spike intervals are either too large (*e.g.*, between the 3rd and the 4th spike) and we deemed them as resting state, or they are in transition from resting state to spiking state, *i.e.*, they are in the ramp state (e.g., the spike interval between the 4th and 5th spike).

3.2 EXAMPLE OF DECODING WITHOUT INTERNAL NOISE SOURCES

We show an example here under noiseless condition. This can be used as a baseline for example in Section 3.3.2 where internal noise sources are present in the neural circuits.

We consider encoding a 0.4 [s] signal bandlimited to 10 [Hz] using neural circuits described in the Supplementary Figure 1, with $M = 4$. That is, a total of 8 neurons are used for encoding. The order of the input space is $L = 4$.

We choose the following feedforward and feedback kernels for neural circuit 1:

$$ {}^1h_1^{111}(t) = 400 \left(\exp\left(-100t\right) \frac{(100t)^3}{3!} - \exp\left(-100t\right) \frac{(100t)^5}{5!} \right), $$

$$ {}^1h_2^{111}(t_1, t_2) = 16(g_c(t_1)g_c(t_2) + g_s(t_1)g_s(t_2)), $$

$$ {}^1h_1^{112}(t) = 0, $$

$$^1h_2^{112}(t_1,t_2) = 16(g_c(t_1)g_c(t_2) - g_s(t_1)g_s(t_2)),$$

$$^1h_1^{221}(t) = 20\exp\left(-200(t-10^{-3})\right)\frac{(200(t-10^{-3}))^3}{3!},$$

$$^1h_1^{212}(t) = 10\exp\left(-200(t-10^{-3})\right)\frac{(200(t-10^{-3}))^3}{3!},$$

The DSP kernels for the rest of three neural circuits uses variations of the above, *e.g.*, different scales (dilations), weights and signs.

We choose a Hodgkin-Huxley neuron in this example. The bias current of all the neurons is set to $10\mu A/cm^2$. We used a simple forward Euler scheme (**Gabbiani and Cox**, 2010) in simulations the Hodgkin-Huxley neuron with integration time step 10^{-6}[s]. The time step is small enough to guarantee the stability and precision of numerical integration. We did not use higher order methods, for example, the staggered Euler scheme (**Hines**, 1984) since the Euler scheme may be more directly comparable to Euler-Maruyama scheme we employed in the stochastic case.

A stimulus is constructed using equation (1) with complex coefficients with real and imaginary parts randomly chosen from a standard normal distribution. A total of 155 spikes are generated from all 8 neurons. Among those 93 are valid measurements. We note that since the t-transform is approximate even under noiseless condition, introducing a smoothing parameters is still necessary. We set $\lambda_1 = \lambda_2 = \lambda$ and used leave-out-one cross validation to find the optimal λ. We leaved out the valid measurements from one neuron each time in the cross validation. We determined the optimal $\lambda = 8.25 \times 10^{-8}$. The reconstruction of $u_1(t)$ is shown in Supplementary Figure 5A. Signal-to-Noise Ratio (SNR) is 25.29 [dB]. The error of the reconstruction of $u_2(t_1,t_2)$ is shown in Supplementary Figure 5E. As suggested, only the part that is sampled by the second order feedforward kernels is recovered. This can be seen from the overlaid kernel on the reconstruction. In particular, $u_1^2(t)$ is recovered as $\hat{u}_2(t,t)$ and the SNR of this recovery is 24.85 [dB] (see Supplementary Figure 5B). By comparison, the SNR of reconstructions without smoothing, *i.e.*, $\lambda = 0$, are 19.14 [dB] for $u_1(t)$ and 8.70 [dB] for $u_1^2(t)$.

Note that in this example, the space $\mathcal{H}_1^1$ is of dimension $2L+1 = 9$, and $\mathcal{H}_2^1$ is of dimension $(2L+1)^2 = 81$. However, since the second order feedforward kernel are all symmetric, they generate a subspace of symmetric functions when sampling $u_2(t_1,t_2)$. This subspace is of dimension $(L+1)(2L+1) = 45$. In addition, each neuron can generate upto $2\cdot 2L+1$ linearly independent sampling functions. Therefore, the minimum number of neurons required for faithfully representing the input stimuli is $(45+9)/(2\cdot 2L+1) = 4$.

3.3 HODGKIN-HUXLEY NEURON WITH CONDUCTANCE NOISE

We constructed the stochastic ion channels using a model of conductance noise rather than the subunit noise used in (30) (**Goldwyn and Shea-Brown**, 2011; **Goldwyn et al.**, 2011). This stochastic Hodgkin-Huxley system is simulated using the diffusion approximation of (**Orio and Soudry**, 2012). The system of SDEs can be expressed as (for clarity, neuron index is not shown)

$$d\mathbf{Y} = \mathbf{f}(\mathbf{Y}, I)dt + \mathbf{B}(\mathbf{Y})d\mathbf{Z}(t),$$

where Y has 14 state variables:

$$\mathbf{Y} = [V, N_0, N_1, N_2, N_3, N_4, M_0H_0, M_1H_0, M_2H_0, M_3H_0, M_0H_1, M_1H_1, M_2H_1, M_3H_1]^T,$$

where $N_i, i = 0, \cdots, 4$, denote the subunit states of potassium channels and $M_iH_j, i = 0, \cdots, 3, j = 0, 1$, denote the stochastic processes modeling the subunit states of sodium channels. The SDE is defined as:

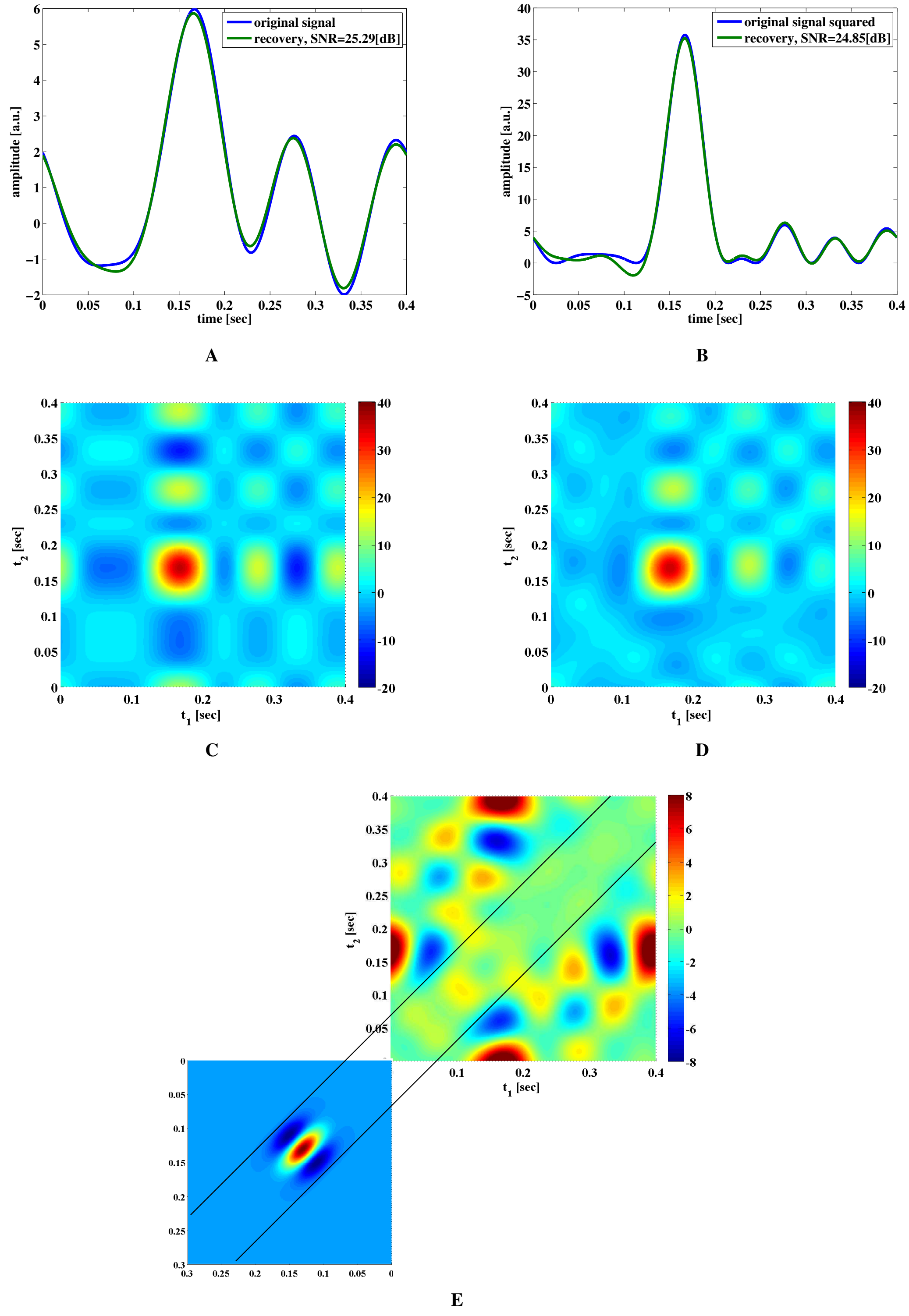

Supplementary Figure 5: Examples of decoding under noiseless condition. (A) Original signal u_1 (blue) and its reconstruction (green). (B) Original $u_1^2(t)$ (blue) and its reconstruction (green). (C) Original $u_2(t_1, t_2) = u_1(t_1)u_1(t_2)$. (D) Reconstruction $\hat{u}_2(t_1, t_2)$. (E) Error between original $u_2(t_1, t_2) = u_1(t_1)u_1(t_2)$ and its reconstruction (top). When evaluating the second order feedforward DSP output, u_2 (in (C)) is multiplied by nonzero values of h_2^{11i} (bottom) only in the domain between the black lines. u_2 in this domain is well reconstructed, whereas it is poorly reconstructed outside of this domain.

$$
\begin{aligned}
dV =& (-\overline{g}_{Na}M_3H_1(V-E_{Na}) - \overline{g}_K N_4(V-E_K) - \overline{g}_L(V-E_L) + I + v)dt + \sigma_1 dW_1 \\
dN_0 =& (-4\alpha_n N_0 + \beta_n N_1)dt + \frac{1}{\sqrt{N_K}}\sqrt{4\alpha_n N_0 + \beta_n N_1}dW_2 \\
dN_1 =& (4\alpha_n N_0 - \beta_n N_1 - 3\alpha_n N_1 + 2\beta_n N_2)dt \\
& - \frac{1}{\sqrt{N_K}}\sqrt{4\alpha_n N_0 + \beta_n N_1}dW_2 + \frac{1}{\sqrt{N_K}}\sqrt{3\alpha_n N_1 + 2\beta_n N_2}dW_3 \\
dN_2 =& (3\alpha_n N_1 - 2\beta_n N_2 - 2\alpha_n N_2 + 3\beta_n N_3)dt \\
& - \frac{1}{\sqrt{N_K}}\sqrt{3\alpha_n N_1 + 2\beta_n N_2}dW_3 + \frac{1}{\sqrt{N_K}}\sqrt{2\alpha_n N_2 + 3\beta_n N_3}dW_4 \\
dN_3 =& (2\alpha_n N_2 - 3\beta_n N_3 - \alpha_n N_3 + 4\beta_n N_4)dt \\
& - \frac{1}{\sqrt{N_K}}\sqrt{2\alpha_n N_2 + 3\beta_n N_3}dW_4 + \frac{1}{\sqrt{N_K}}\sqrt{\alpha_n N_3 + 4\beta_n N_4}dW_5 \\
dN_4 =& (\alpha_n N_3 - 4\beta_n N_4)dt - \frac{1}{\sqrt{N_K}}\sqrt{\alpha_n N_3 + 4\beta_n N_4}dW_5 \\
dM_0H_0 =& (-3\alpha_m M_0H_0 + \beta_m M_1H_0 - \alpha_h M_0H_0 + \beta_h M_0H_1)dt \\
& + \frac{1}{\sqrt{N_{Na}}}\sqrt{3\alpha_m M_0H_0 + \beta_m M_1H_0}dW_6 + \frac{1}{\sqrt{N_{Na}}}\sqrt{\alpha_h M_0H_0 + \beta_h M_0H_1}dW_9 \\
dM_1H_0 =& (3\alpha_m M_0H_0 - \beta_m M_1H_0 - 2\alpha_m M_1H_0 + 2\beta_m M_2H_0 - \alpha_h M_1H_0 + \beta_h M_1H_1)dt \\
& - \frac{1}{\sqrt{N_{Na}}}\sqrt{3\alpha_m M_0H_0 + \beta_m M_1H_0}dW_6 + \frac{1}{\sqrt{N_{Na}}}\sqrt{2\alpha_m M_1H_0 + 2\beta_m M_2H_0}dW_7 \\
& + \frac{1}{\sqrt{N_{Na}}}\sqrt{\alpha_h M_1H_0 + \beta_h M_1H_1}dW_{10} \\
dM_2H_0 =& (2\alpha_m M_1H_0 - 2\beta_m M_2H_0 - \alpha_m M_2H_0 + 3\beta_m M_3H_0 - \alpha_h M_2H_0 + \beta_h M_2H_1)dt \\
& - \frac{1}{\sqrt{N_{Na}}}\sqrt{2\alpha_m M_1H_0 + 2\beta_m M_2H_0}dW_7 + \frac{1}{\sqrt{N_{Na}}}\sqrt{\alpha_m M_2H_0 + 3\beta_m M_3H_0}dW_8 \\
& + \frac{1}{\sqrt{N_{Na}}}\sqrt{\alpha_h M_2H_0 + \beta_h M_2H_1}dW_{11} \\
dM_3H_0 =& (\alpha_m M_2H_0 - 3\beta_m M_3H_0 - \alpha_h M_3H_0 + \beta_h M_3H_1)dt \\
& - \frac{1}{\sqrt{N_{Na}}}\sqrt{\alpha_m M_2H_0 + 3\beta_m M_3H_0}dW_8 + \frac{1}{\sqrt{N_{Na}}}\sqrt{\alpha_h M_3H_0 + \beta_h M_3H_1}dW_{12} \\
dM_0H_1 =& (-3\alpha_m M_0H_1 + \beta_m M_1H_1 + \alpha_h M_0H_0 - \beta_h M_0H_1)dt \\
& + \frac{1}{\sqrt{N_{Na}}}\sqrt{3\alpha_m M_0H_1 + \beta_m M_1H_1}dW_{13} - \frac{1}{\sqrt{N_{Na}}}\sqrt{\alpha_h M_0H_0 + \beta_h M_0H_1}dW_9
\end{aligned}
$$

$$
\begin{aligned}
dM_1H_1 =&(3\alpha_m M_0H_1 - \beta_m M_1H_1 - 2\alpha_m M_1H_1 + 2\beta_m M_2H_1 + \alpha_h M_1H_0 - \beta_h M_1H_1)dt \\
&- \frac{1}{\sqrt{N_{Na}}}\sqrt{3\alpha_m M_0H_1 + \beta_m M_1H_1}dW_{13} + \frac{1}{\sqrt{N_{Na}}}\sqrt{2\alpha_m M_1H_1 + 2\beta_m M_2H_1}dW_{14} \\
&- \frac{1}{\sqrt{N_{Na}}}\sqrt{\alpha_h M_1H_0 + \beta_h M_1H_1}dW_{10} \\
dM_2H_1 =&(2\alpha_m M_1H_1 - 2\beta_m M_2H_1 - \alpha_m M_2H_1 + 3\beta_m M_3H_1 + \alpha_h M_2H_0 - \beta_h M_2H_1)dt \\
&- \frac{1}{\sqrt{N_{Na}}}\sqrt{2\alpha_m M_1H_1 + 2\beta_m M_2H_1}dW_{14} + \frac{1}{\sqrt{N_{Na}}}\sqrt{\alpha_m M_2H_1 + 3\beta_m M_3H_1}dW_{15} \\
&- \frac{1}{\sqrt{N_{Na}}}\sqrt{\alpha_h M_2H_0 + \beta_h M_2H_1}dW_{11} \\
dM_3H_1 =&(\alpha_m M_2H_1 - 3\beta_m M_3H_1 + \alpha_h M_3H_0 - \beta_h M_3H_1)dt \\
&- \frac{1}{\sqrt{N_{Na}}}\sqrt{\alpha_m M_2H_1 + 3\beta_m M_3H_1}dW_{15} - \frac{1}{\sqrt{N_{Na}}}\sqrt{\alpha_h M_3H_0 + \beta_H M_3H_1}dW_{12}
\end{aligned}
$$

where $\alpha_n = \alpha_n(V), \beta_n = \beta_n(V), \alpha_m = \alpha_m(V), \beta_m = \beta_m(V), \alpha_h = \alpha_h(V), \beta_h = \beta_h(V)$ are defined as in the standard form (S2) and N_K, N_{Na} are the numbers of potassium and sodium channels, respectively.

4 SUPPLEMENTARY MATERIAL FOR SECTION 4

4.1 EXAMPLE OF IDENTIFICATION UNDER NOISELESS CONDITION

We provide here an example of functional identification of the neuron circuit 1 of Supplementary Section 3.2 under noiseless condition; the same DSP kernels and Hodgkin-Huxley neuron model were used.

First, we use the circuit to encode a 50 [Hz] input signals of duration 0.4 [s]. We repeat this for $1,000$ times with a different, randomly generated input each time.

In the $1,000$ trials, a total of $18,964$ spikes are generated by BSG 1 and $25,271$ spikes are generated by BSG 2. We deem the maximum valid interspike interval to be 13.713[msec]. Among all the spikes, valid measurements amount to $9,615$ for BSG 1 and $8,093$ for BSG 2. The identified DSP kernels are shown in Supplementary Figure 6. We also show identification quality against the number of valid measurements in Figure 6I. The identification quality saturates after using more than $2,000$ measurements. This correspond to about 200 trials.

The identification results for the DSP kernels associated with Neuron 2 are shown in Supplementary Figure 7.

4.2 EXAMPLE OF IDENTIFICATION WITH INTEGRATE-AND-FIRE NEURON

We also provide here an example using Integrate-and-Fire (IAF) Neurons instead of using Hodgkin-Huxley neurons as in Supplementary Section 4.1. Since the t-transforms of encoding with IAF neurons are exact, we show here that the identification algorithm leads to perfect identification when appropriate input stimuli and spike spaces are used.

Supplementary Figure 8 shows the identification result for neuron 1 using the same input stimulus spaces and spike spaces as in Supplementary Section 4.1.

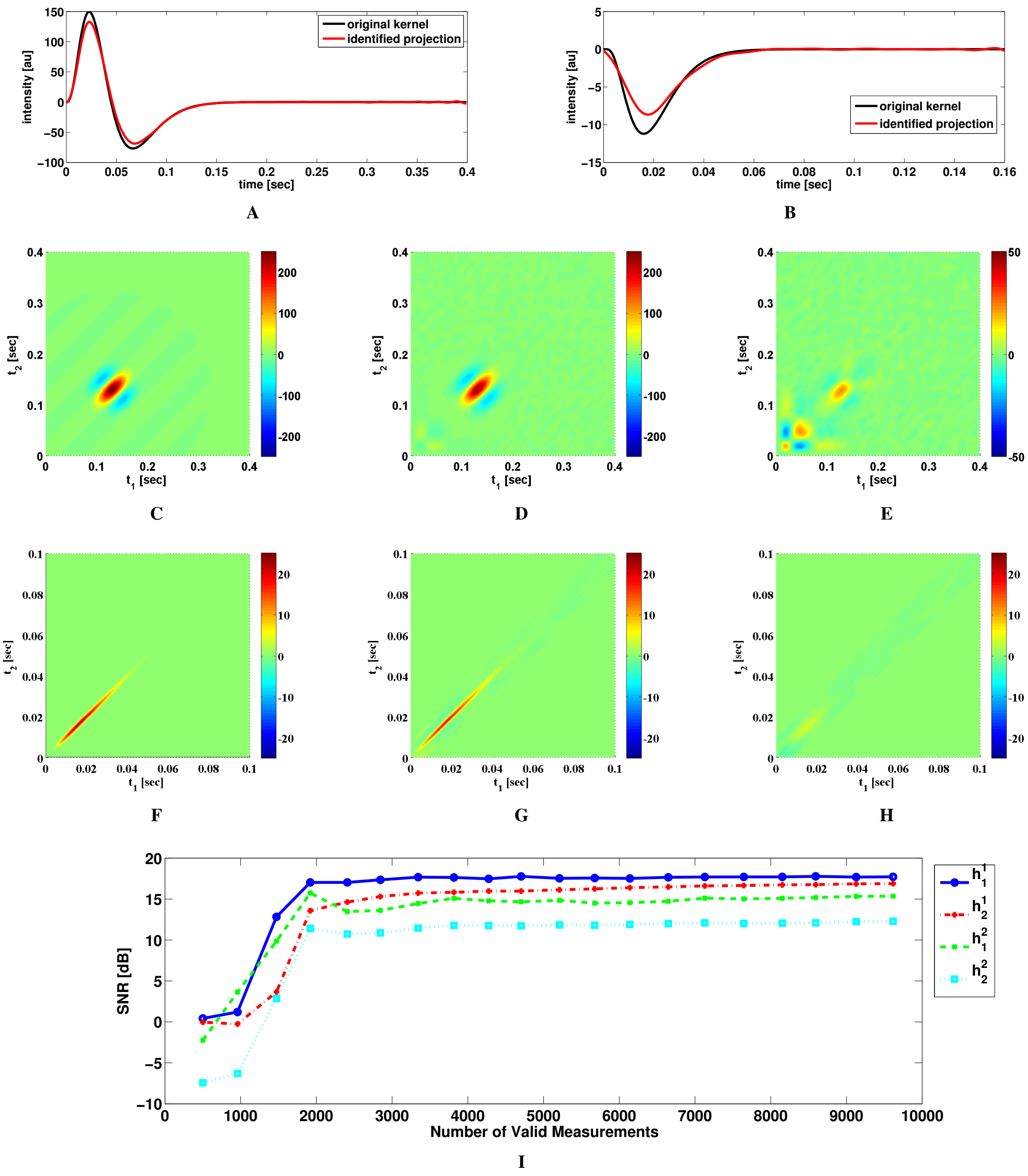

Supplementary Figure 6: Examples of functional identification of a neural circuit with Hodgkin-Huxley neurons as spike generators under noiseless condition (Neuron 1). (A) Original first order feedforward kernel (black) and identified projection of the kernel (red). (B) Original first order feedback kernel (black) and identified projection of the kernel (red). (C) Original second order feedforward kernel. (D) Identified projection of second order feedforward kernel. (E) Error of identified second order feedforward kernel. (F) Original second order feedback kernel. (G) Identified projection of second order feedback kernel. (H) Error of identified second order feedback kernel. (I) SNR of the identified DSP kernels that feed into Neuron 1 against number of valid spikes used in identification.

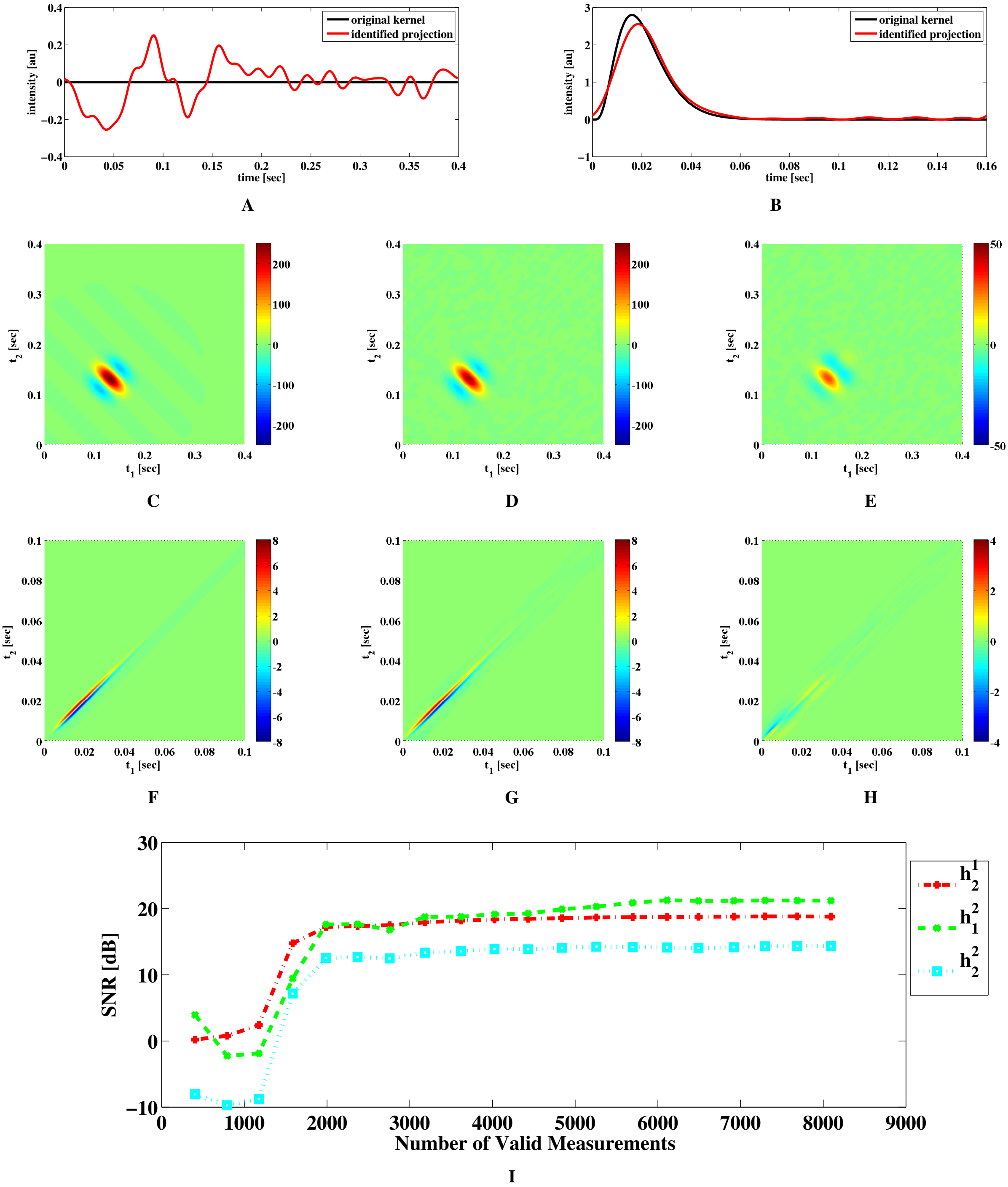

Supplementary Figure 7: Examples of functional identification of a neural circuit with Hodgkin-Huxley neurons as spike generators under noiseless condition (Neuron 2). (A) Original first order feedforward kernel (black) and identified projection of the kernel (red). Note that the original first order kernel is zero. (B) Original first order feedback kernel (black) and identified projection of the kernel (red). (C) Original second order feedforward kernel. (D) Identified projection of second order feedforward kernel. (E) Error of identified second order feedforward kernel. (F) Original second order feedback kernel. (G) Identified projection of second order feedback kernel. (H) Error of identified second order feedback kernel. (I) SNR of the identified DSP kernels that feed into Neuron 2 against number of valid spikes used in identification. h_1^1 is omitted since it is zero.

Supplementary Figure 9 shows the identification result for neuron 1 using a higher bandwidth for the spike space when compared with the one used in Supplementary Figure 8.

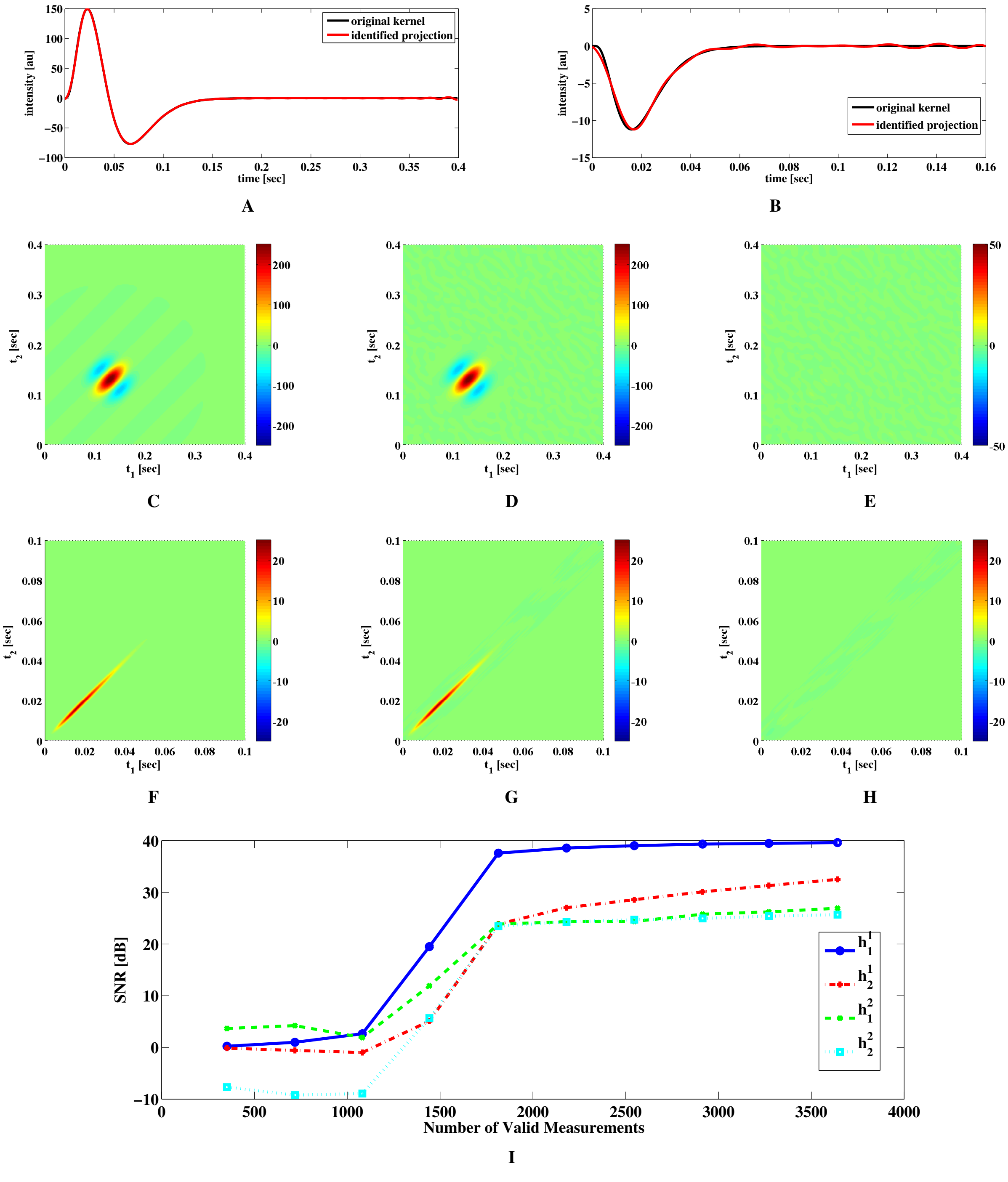

Supplementary Figure 8: Examples of functional identification of a neural circuit with IAF neurons as spike generators under noiseless condition (Neuron 1). The IAF neurons has a refractory period of 2 [msec]. (A) Original first order feedforward kernel (black) and identified projection of the kernel (red). (B) Original first order feedback kernel (black) and identified projection of the kernel (red). (C) Original second order feedforward kernel. (D) Identified projection of second order feedforward kernel. (E) Error of identified second order feedforward kernel. (F) Original second order feedback kernel. (G) Identified projection of second order feedback kernel. (H) Error of identified second order feedback kernel. (I) SNR of the identified DSP kernels that feed into Neuron 1 against number of valid spikes used in identification.

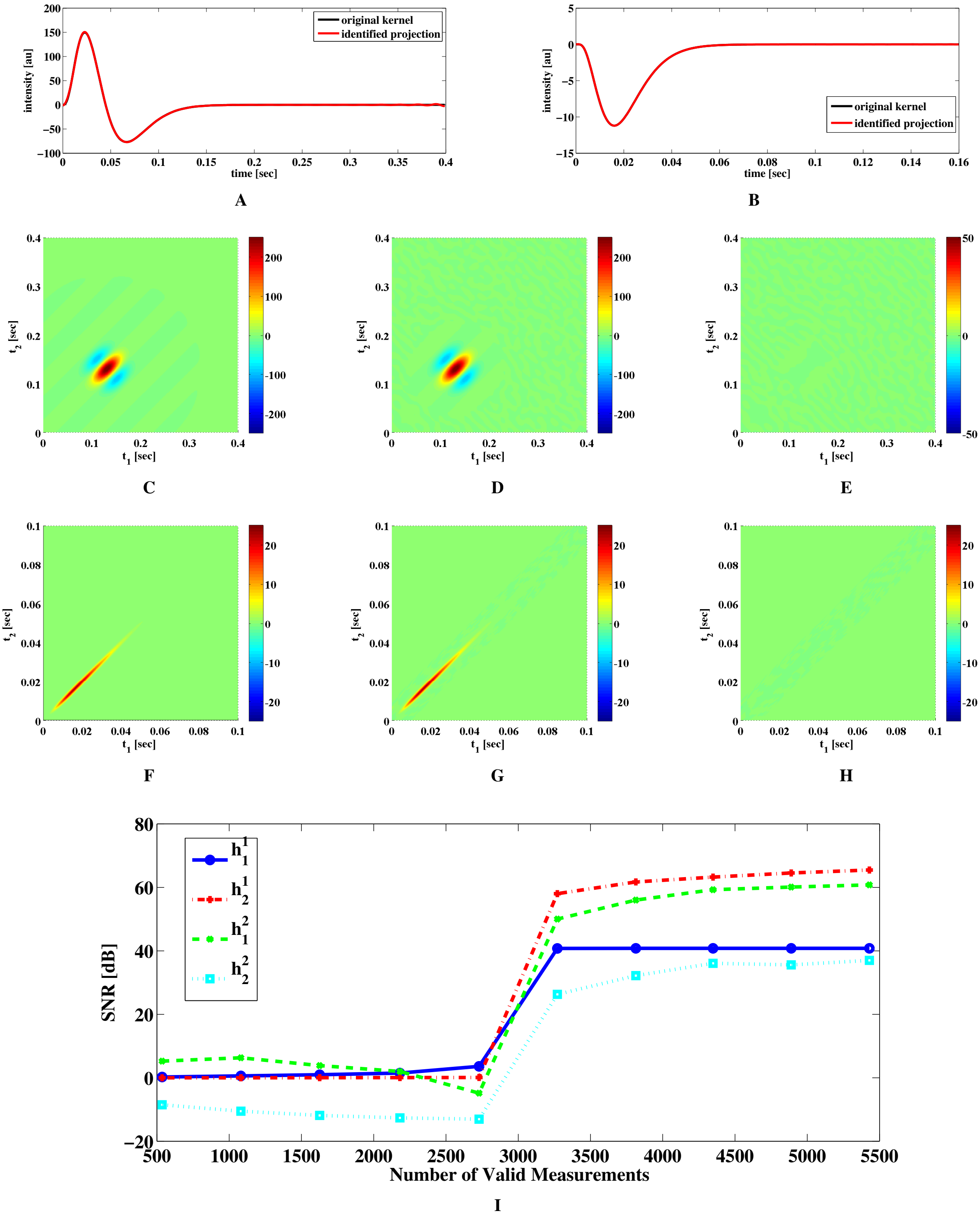

Supplementary Figure 9: Examples of functional identification of a neural circuit with IAF neurons as spike generators under noiseless condition (Neuron 1). The IAF neurons has a refractory period of 2 [msec]. Compared to the example in Supplementary Figure 8, we increased the bandwidth of the space $\mathcal{H}_1^2$, *i.e.*, the space of spikes, to better approximate the effect of feedback spikes on the dendritic current. One can see that the quality of the identified feedback kernels further increases. So does the quality of the identified feedforward kernels. The sum of the orders of the kernel spaces is 2982. (A) Original first order feedforward kernel (black) and identified projection of the kernel (red). (B) Original first order feedback kernel (black) and identified projection of the kernel (red). (C) Original second order feedforward kernel. (D) Identified projection of second order feedforward kernel. (E) Error of identified second order feedforward kernel. (F) Original second order feedback kernel. (G) Identified projection of second order feedback kernel. (H) Error of identified second order feedback kernel. (I) SNR of the identified DSP kernels that feed into Neuron 1 against number of valid spikes used in identification.

REFERENCES

Gabbiani, F. and Cox, S. J. (2010), Mathematics For Neuroscientists (Academic Press)

Goldwyn, J. H., Imennov, N. S., Famulare, M., and Shea-Brown, E. (2011), Stochastic differential equation models for ion channel noise in hodgkin-huxley neurons, *Physics Review E*, 83, 4, 041908, doi:10.1103/PhysRevE.83.041908

Goldwyn, J. H. and Shea-Brown, E. (2011), The what and where of adding channel noise to the hodgkin-huxley equations, *PLoS Computational Biology*, 7, 11, e1002247, doi:10.1371/journal.pcbi.1002247

Hines, M. (1984), Efficient computation of branched nerve equations, *International Journal of Bio-Medical Computing*, 15, 1, 69–76

Izhikevich, E. M. (2007), Dynamical Systems in Neuroscience: The Geometry of Excitability and Bursting (MIT Press, Cambridge, MA)

Lazar, A. A. (2010), Population encoding with hodgkin-huxley neurons, *IEEE Transactions on Information Theory*, 56, 2, 821–837, doi:10.1109/TIT.2009.2037040, special Issue on Molecular Biology and Neuroscience

Orio, P. and Soudry, D. (2012), Simple, fast and accurate implementation of the diffusion approximation algorithm for stochastic ion channels with multiple states, *PLoS One*, 7, 5, e36670, doi:10.1371/journal.pone.0036670
